# *In vitro* activity of cefiderocol against European *Pseudomonas aeruginosa* and *Acinetobacter* spp., including isolates resistant to meropenem and recent β-lactam/β-lactamase inhibitor combinations

**DOI:** 10.1128/spectrum.03836-23

**Published:** 2024-03-14

**Authors:** Anne Santerre Henriksen, Katy Jeannot, Antonio Oliver, John D. Perry, Mathias W. Pletz, Stefania Stefani, Ian Morrissey, Christopher Longshaw, ARTEMIS Study Investigators, Birgit Willinger

**Affiliations:** 1 Medical Affairs, Shionogi B.V., London, United Kingdom; 2 Laboratory of Bacteriology, University Hospital of Besançon, University of Franche-Comté, Besançon, France; 3 Servicio de Microbiología and Unidad de Investigación, Hospital Universitario Son Espases, Instituto de Investigación Sanitaria Illes Balears (IdISBa), Centro de Investigación Biomédica en Red en Enfermedades Infecciosas (CIBERINFEC), Palma de Mallorca, Spain; 4 Microbiology Department, Freeman Hospital, Newcastle upon Tyne, United Kingdom; 5 Institute of Infectious Diseases and Infection Control, Jena University Hospital, Jena, Germany; 6 Department of Biomedical and Biotechnological Sciences, University of Catania, Catania, Italy; 7 Antimicrobial Focus Ltd., Sawbridgeworth, United Kingdom; Helmholtz-Institut fur Pharmazeutische Forschung Saarland, Saarbrücken, Germany

**Keywords:** cefiderocol, *Pseudomonas aeruginosa*, *Acinetobacter *spp., β‍-‍l‍a‍c‍t‍a‍m‍/‍β‍-‍l‍a‍‍c‍t‍a‍m‍a‍s‍e inhibitor combinations, meropenem, ceftazidime-avibactam, ceftolozane-tazobactam, meropenem-vaborbactam, imipenem-relebactam, a‍z‍t‍r‍e‍o‍n‍a‍m‍-‍a‍v‍i‍b‍a‍c‍t‍a‍m, cefepime-taniborbactam, sulbactam-durlobactam, resistance, meropenem-resistant, β-lactamases, Europe, *in vitro*

## Abstract

Carbapenem-resistant *Pseudomonas aeruginosa* and *Acinetobacter* spp. represent major threats and have few approved therapeutic options. Non-‍fermenting Gram-negative isolates were collected from hospitalized inpatients from 49 sites in 6 European countries between 01 January 2020 and 31 December 2020 and underwent susceptibility testing against cefiderocol and β-lactam/β-lactamase inhibitor combinations. Meropenem-resistant (MIC >8 mg/L), cefiderocol-susceptible isolates were analyzed by PCR, and cefiderocol-resistant isolates were analyzed by whole-genome sequencing to identify resistance mechanisms. Overall, 1,451 (950 *P*. *aeruginosa*; 501 *Acinetobacter* spp.) isolates were collected, commonly from the respiratory tract (42.0% and 39.3%, respectively). Cefiderocol susceptibility was higher than ‍β‍-‍l‍a‍c‍t‍a‍m‍/‍β‍-‍l‍a‍c‍t‍a‍mase‍ inhibitor combinations against *P. aeruginosa* (98.9% vs 83.3%–91.4%), and *P. ‍aeruginosa* resistant to meropenem (*n* = 139; 97.8% vs 12.2%–59.7%), β-lactam/β-lactamase inhibitor combinations (93.6%–98.1% vs 10.7%–71.8%), and both meropenem and ceftazidime-avibactam (96.7% vs 5.0%–‍‍45.0%) or ‍ceftolozane-tazobactam (98.4% vs 8.1%–54.8%), respectively. Cefiderocol and sulbactam-durlobactam susceptibilities were high against *Acinetobacter* spp. (92.4% and 97.0%) and meropenem-resistant *Acineto‍bacter* ‍spp. (*n* = 227; 85.0% and 93.8%) but lower against sulbactam-durlobactam- (*n* ‍= 15; 13.3%) and cefiderocol- (*n* = 38; 65.8%) resistant isolates, respectively. Among meropenem-resistant *P. aeruginosa* and *Acinetobacter* spp., the most common β-‍‍lactamase genes were metallo-β-lactamases [30/139; *bla*_VIM-2_ (15/139)] and oxacillinases [215/227; *bla*_OXA-23_ (194/227)], respectively. Acquired β-lactamase genes were identified in 1/10 and 32/38 of cefiderocol-resistant *P. aeruginosa* and *Acinetobacter* spp., and *pirA*-like or *piuA* mutations in 10/10 and 37/38, respectively. *Conclusion:* cefiderocol susceptibility was high against *P. aeruginosa* and *Acinetobacter* spp., including meropenem-resistant isolates and those resistant to recent β-lactam/β-lactamase inhibitor combinations common in first-line treatment of European non-fermenters.

**IMPORTANCE:**

This was the first study in which the *in vitro* activity of cefiderocol and non-licensed β-lactam/β-lactamase inhibitor combinations were directly compared against *Pseudomonas aeruginosa* and *Acinetobacter* spp., including meropenem- and β-lactam/β-lactamase inhibitor combination-resistant isolates. A notably large number of European isolates were collected. Meropenem resistance was defined according to the MIC breakpoint for high-dose meropenem, ensuring that data reflect antibiotic activity against isolates that would remain meropenem resistant in the clinic. Cefiderocol susceptibility was high against non-fermenters, and there was no apparent cross resistance between cefiderocol and β-lactam/β-lactamase inhibitor combinations, with the exception of sulbactam-durlobactam. These results provide insights into therapeutic options for infections due to resistant *P. aeruginosa* and *Acinetobacter* spp. and indicate how early susceptibility testing of cefiderocol in parallel with β-lactam/β-lactamase inhibitor combinations will allow clinicians to choose the effective treatment(s) from all available options. This is particularly important as current treatment options against non-fermenters are limited.

## INTRODUCTION

Antimicrobial resistance is widespread throughout Europe (1). In particular, the high rates of carbapenem resistance observed in *Pseudomonas aeruginosa* and *Acineto‍bacter* ‍spp. (19% and 48%, respectively, in 2021) represent a major threat, considering few approved therapeutic options are available (1, 2). The World Health Organization has, therefore, recognized carbapenem-resistant (CR) *P. aeruginosa* and CR *‍Acinetobacter ‍baumannii* (CRAB) as critical priority pathogens (2).

Major mechanisms of carbapenem resistance in *P. aeruginosa* include loss of outer membrane porin D function, overexpression of efflux pumps, and carbapenemases [including Class B metallo-β-lactamases (MBLs) such as the Verona integron-borne MBL (VIM) and emerging class A carbapenemases such as Guiana extended-spectrum β-lactamase (GES) and *Klebsiella pneumoniae* carbapenemase (KPC)] (3–9). Carbapenemases most commonly found in CR ‍*Acinetobacter* spp. include OXA-23 and OXA-24/40 oxacillinases; with the exception of New Delhi MBL (NDM)-1, MBLs are less common, although their potency makes them problematic for treatment (5, 10–12). Efflux pumps and reduced membrane permeability due to CarO porin loss are also associated with carbapenem resistance in *Acinetobacter* spp. (13, 14).

Cefiderocol is a unique catechol-siderophore cephalosporin approved in Europe for the treatment of infections due to aerobic Gram-negative organisms in adults with limited treatment options (15). The mechanism of action of cefiderocol is the disruption of peptidoglycan cell-wall synthesis via inhibition of penicillin-binding proteins (PBPs) (16, 17). The structure of cefiderocol and “Trojan Horse” mechanism of bacterial cell entry provide enhanced stability to a wide range of β-lactamases, and allow for the activity of cefiderocol to be broadly unaffected by efflux pump overexpression and porin channel modifications observed in CR non-fermenters (17–21).

Various β-lactam/β-lactamase inhibitor (BLBLI) combinations have been approved (ceftazidime-avibactam, ceftolozane-tazobactam, imipenem-relebactam, and meropenem-vaborbactam) or are in development (aztreonam-avibactam, c‍e‍f‍e‍p‍i‍m‍e‍-‍t‍a‍n‍i‍b‍o‍r‍b‍a‍c‍t‍a‍m, and sulbactam-durlobactam) for clinical use in Europe to treat infections caused by CR *P. aeruginosa* and/or *Acinetobacter* spp. (22–27). However, several of these combinations are affected or rendered inactive by one or more of the known mechanisms of resistance in CR non-fermenters; these BLBLI combinations remain unable to inhibit certain β-lactamases, MBLs in particular, and are still affected by porin channel modifications (28–32).

There is notable variation in the European Society of Clinical Microbiology and Infectious Diseases and Infectious Diseases Society of America guidance for treatment of infections due to CR *P. aeruginosa* and *Acinetobacter* spp., although both generally recognize that *in vitro* activity of antimicrobials is an important consideration for treatment decision-making (33, 34). The longitudinal surveillance studies SENTRY and SIDERO generated data on the susceptibilities of cefiderocol and approved BLBLI combinations against non-fermenters, including CR and BLBLI combination-resistant isolates (35, 36). Although, despite the current and anticipated use of cefiderocol and BLBLI combinations against CR Gram-negative infections, there remains limited data comparing these antimicrobials and developmental BLBLI combinations against CR non-fermenter isolates, including those also resistant to a comparator antimicrobial.

The aim of this study was to evaluate the *in vitro* activity of cefiderocol, meropenem, BLBLI combinations (approved and in development), and colistin against clinical Gram-negative isolates collected between 01 January 2020 and 31 December 2020, across six countries in Europe. Here, we report the results for *P. aeruginosa* and *Acine‍tobacter* ‍spp. isolates. Results for Enterobacterales isolates collected in this study are reported elsewhere.

## RESULTS

### Epidemiology

In total, 1,451 isolates were collected from European hospitals, of which 950 (65.5%) were *P. aeruginosa* and 501 (34.5%) were *Acinetobacter* spp. [including 458 (91.4%) *A*. ‍*baumannii* complex] (see Table S1 for isolates by country). *P. aeruginosa* and *A‍c‍i‍‍n‍e‍t‍o‍b‍a‍c‍t‍e‍r* ‍spp. isolates were collected from a range of infection sources, the most common for both species being respiratory tract [42.0% (399/950); 39.3% (197/501)], bloodstream [25.4% (241/950); 26.3% (132/501)], and skin [21.7% (206/950); 20.0% (100/501)], respectively (see Fig. S1 for all infection sources).

### Susceptibility profiles of isolates

Susceptibility to cefiderocol was 98.9% against *P. aeruginosa* isolates and 92.4% against *Acinetobacter* spp. isolates [Table 1; see Table S2 for susceptibility rates using Clinical and Laboratory Standards Institute (CLSI) breakpoints or United States Food and Drug Administration (FDA) breakpoints where CLSI breakpoints were not available]. By individual country, susceptibility to cefiderocol was similar against *P. aeruginosa*, ranging from 98.7% in France to 99.3% in the United Kingdom, but showed more variability against *Acinetobacter* spp. (88.7% in Italy to 98.0% in Spain) (Table S3A through E). Against *P. aeruginosa* isolates, rates of susceptibility to BLBLI combinations were lower than cefiderocol, ranging from 83.3% for imipenem-relebactam to 91.4% for c‍e‍f‍e‍p‍i‍m‍e‍-‍t‍a‍n‍i‍b‍o‍r‍b‍a‍c‍t‍a‍m (Table 1). Against *Acinetobacter* spp. isolates, rates of susceptibility were 92.4% to cefiderocol and 97.0% to sulbactam-durlobactam (FDA breakpoint) (Table 1) (37, 38). Using epidemiological cut-off (ECOFF) values, susceptibility to colistin was 99.7% and 98.4% for *P. aeruginosa* and *Acinetobacter* ‍spp., respectively (Table 1).

**TABLE 1:** *In vitro* activity of cefiderocol, BLBLI combinations, and other relevant antibiotics against *P. aeruginosa* and *Acinetobacter* spp. isolates^*a*^^,^^*b*^

	FDC		MEM		CZA		C-T		MVB		I-R		ATM-AVI		FEP-TAN		SUL-DUR		(CST)	
Isolates (*n*)	MIC_90_ (mg/L)	*S* (%)	MIC_90_ (mg/L)	*S*/*I* (%)	MIC_90_ (mg/L)	*S* (%)	MIC_90_ (mg/L)	*S* (%)	MIC_90_ (mg/L)	*S*/*I* (%)	MIC_90_ (mg/L)	*S* (%)	MIC_90_ (mg/L)	*S*/*I* (%)	MIC_90_ (mg/L)	*S*/*I* (%)	MIC_90_ (mg/L)	*S* (%)	MIC_90_ (mg/L)	*S* (%)
*P. aeruginosa* (950)	1	98.9	16	85.4	8	90.1	8	89.1	16	87.2	4	83.3	32	86.2	8	91.4	N/A	N/A	(1)	(99.7)
*Acinetobacter* spp. (501)^*c*^	2	92.4	>16	54.7	N/A	N/A	N/A	N/A	N/A	N/A	N/A	N/A	N/A	N/A	N/A	N/A	4	97.0	(0.5)	(98.4)

^*a*^ ATM-AVI, aztreonam-avibactam; BLBLI, β-lactam/β-lactamase inhibitor; CST, colistin; C-T, ceftolozane-tazobactam; CZA, ceftazidime-avibactam; ECOFF, epidemiological cut-off; EUCAST, European Committee on Antimicrobial Susceptibility Testing; FDA, Food and Drug Administration; FDC, cefiderocol; FEP-TAN, cefepime-taniborbactam; I, susceptible, increased exposure; I-R, imipenem-relebactam; MEM, meropenem; MVB, meropenem-vaborbactam; N/A, not applicable; PD, pharmacodynamic; PK, pharmacokinetic; S, susceptibility; SUL-DUR, sulbactam-durlobactam.

^*b*^ Antibiotics were tested against *P. aeruginosa* and/or *Acinetobacter* spp. based on expected use in a real-world setting. Susceptibility was assessed according to EUCAST breakpoints (including non-species-specific PK/PD breakpoints, high-dosage breakpoints, and breakpoints for the agent without inhibitor, where applicable), except for sulbactam-durlobactam and colistin where FDA breakpoints and ECOFF values were used, respectively. Data on susceptibility to colistin are shown in parentheses as colistin is not recommended for monotherapy and is not associated with a clinical monotherapy breakpoint (as per EUCAST v.14.0 guidance).

^*c*^ Includes 458 *A. baumannii* complex isolates.

### Susceptibility profiles of isolates with antibiotic-resistant phenotypes

**Fig 1:**
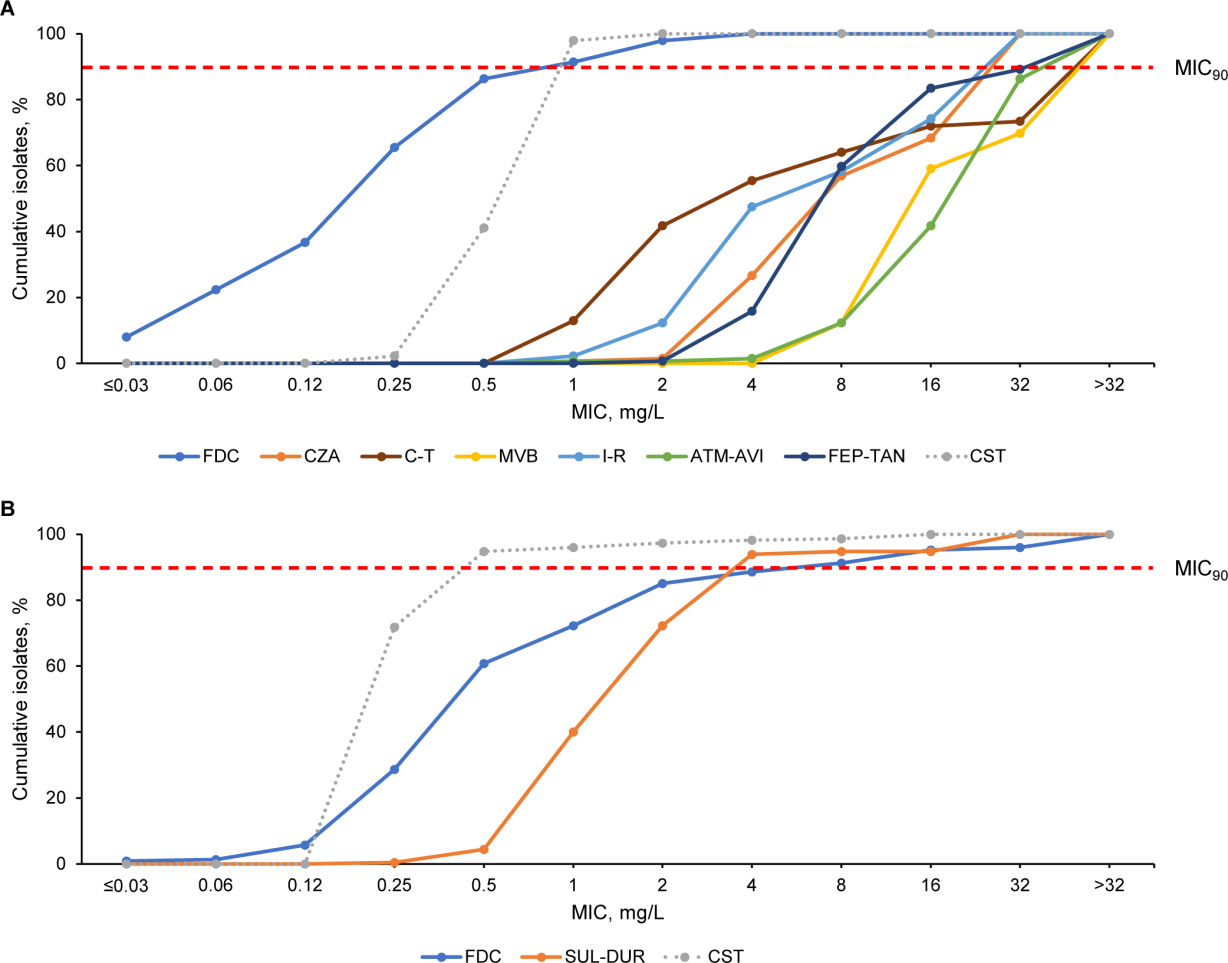
Cumulative MIC distributions of cefiderocol, BLBLI combinations, and colistin against (**A**) meropenem-resistant *P. aeruginosa* (*n* = 139) and (**B**) meropenem-resistant *Acinetobacter* spp. (*n* = 227). Antimicrobials were tested against *P. aeruginosa* and/or *Acinetobacter* spp. based on expected use in a real-world setting. Data on susceptibility to colistin are shown as dashed lines as colistin is not recommended for monotherapy and is not associated with a clinical monotherapy breakpoint (as per EUCAST v.14.0 guidance). Resistance to meropenem was defined using a breakpoint of MIC >8 mg/L, relating to high-dose, extended-infusion (2 g, 3-h infusion) meropenem (as per EUCAST v14.0 guidance). ATM-AVI, aztreonam-avibactam; BLBLI, β-lactam/β-lactamase inhibitor; CST, colistin; C-T, ceftolozane-tazobactam; CZA, ceftazidime-avibactam; EUCAST, European Committee on Antimicrobial Susceptibility Testing; FDC, cefiderocol; FEP-TAN, cefepime-taniborbactam; I-R, imipenem-relebactam; MVB, meropenem-vaborbactam; SUL-DUR, sulbactam-durlobactam.

In total, 14.6% (139/950) *P*. *aeruginosa* isolates were classified as meropenem resistant (meropenem MIC >8 mg/L) (Table 2; see Fig. 1A for MIC distributions). Susceptibility of meropenem-resistant *P. aeruginosa* to cefiderocol was 97.8%, which was higher than susceptibility rates of <60% to all tested BLBLI combinations (Table 2). M‍e‍r‍o‍p‍e‍n‍e‍m‍-‍v‍a‍b‍o‍r‍b‍a‍c‍t‍a‍m and imipenem-relebactam had the lowest susceptibility (12.2%), followed by aztreonam-‍avibactam (41.7%) and then cefepime-‍‍taniborbactam, ceftazidime-‍‍avibactam, and ceftolozane-tazobactam (55.4%–59.7%) (Table 2). This trend was detected across all countries (Table S4A through ‍D).

**TABLE 2:** *In vitro* activity of cefiderocol, BLBLI combinations, and other relevant antibiotics against *P. aeruginosa* and *Acinetobacter* spp. isolates with resistant phenotypes^*a*^^,^^*b*^

Isolates	*n*	Susceptibility^*c*^ (%)									
		FDC	MEM	CZA	C-T	MVB	I-R	ATM-AVI	FEP-TAN	SUL-DUR	(CST)
*P. aeruginosa*	950	98.9	85.4	90.1	89.1	87.2	83.3	86.2	91.4	N/A	(99.7)
MEM-R	139	97.8		56.8	55.4	12.2	12.2	41.7	59.7	N/A	(100)
CZA-R	94	93.6	36.2		36.2	39.4	37.2	54.3	55.3	N/A	(100)
C-T-R	104	94.2	40.4	42.3		45.2	36.5	64.4	59.6	N/A	(100)
MVB-R	122	97.5	0	53.3	53.3		10.7	38.5	59.0	N/A	(100)
I-R-R	159	98.1	23.3	62.9	58.5	31.4		54.1	64.8	N/A	(100)
ATM-AVI-R	131	95.4	38.2	67.2	71.8	42.7	44.3		61.8	N/A	(100)
FEP-TAN-R	82	95.1	31.7	48.8	48.8	39.0	31.7	39.0		N/A	(100)
MEM-R and CZA-R	60	96.7			28.3	5.0	8.3	45.0	43.3	N/A	(100)
MEM-R and C-T-R	62	98.4		30.6		8.1	9.7	54.8	50.0	N/A	(100)
*Acinetobacter* spp.	501^*d*^	92.4	54.7	N/A	N/A	N/A	N/A	N/A	N/A	97.0	(98.4)
FDC-R	38		10.5	N/A	N/A	N/A	N/A	N/A	N/A	65.8	(92.1)
MEM-R	227	85.0		N/A	N/A	N/A	N/A	N/A	N/A	93.8	(97.4)
SUL-DUR-R^*e*^	15	13.3	6.7	N/A	N/A	N/A	N/A	N/A	N/A		(100)

^*a*^ ATM-AVI, aztreonam-avibactam; BLBLI, β-lactam/β-lactamase inhibitor; CST, colistin; C-T, ceftolozane-tazobactam; CZA, ceftazidime-avibactam; ECOFF, epidemiological cut-off; EUCAST, European Committee on Antimicrobial Susceptibility Testing; FDA, Food and Drug Administration; FDC, cefiderocol; FEP-TAN, cefepime-taniborbactam; I-R, imipenem-relebactam; MEM, meropenem; MVB, meropenem-vaborbactam; N/A, not applicable; PD, T, ceftolozane-tazobactam; CZA, ceftazidime-avibactam; ECOFF, epidemiological cut-off; EUCAST, European Committee on Antimicrobial Susceptibility Testing; FDA, Food and Drug Administration; FDC, cefiderocol; FEP-TAN, cefepime-taniborbactam; I-R, imipenem-relebactam; MEM, meropenem; MVB, meropenem-vaborbactam; N/A, not applicable; PD, pharmacodynamic; PK, pharmacokinetic; R, resistant; SUL-‍‍DUR, sulbactam-durlobactam.

^*b*^ Antimicrobials were tested against *P. aeruginosa* and/or *Acinetobacter* spp. based on expected use in a real-world setting. Results are not reported for isolates tested against antibiotics to which they had an expected resistance phenotype. Susceptibility was assessed according to EUCAST breakpoints (including non-species-specific PK/PD breakpoints, high dosage breakpoints, and breakpoints for the agent without inhibitor, where applicable), except for sulbactam-durlobactam and colistin where FDA breakpoints and ECOFF values were used, respectively. Data are shown where *n* ≥ 20 isolates were available. Data on susceptibility to colistin are shown in parentheses as colistin is not recommended for monotherapy and is not associated with a clinical monotherapy breakpoint (as per EUCAST v.14.0 guidance).

^*c*^ Refers to susceptibility, or susceptibility with increased exposure for meropenem, meropenem-vaborbactam, aztreonam-avibactam, and cefepime-taniborbactam.

^*d*^ Includes 458 *A. baumannii* complex isolates.

^*e*^ Sulbactam-durlobactam-resistant isolates were included irrespective of the number of isolates due to the scarcity of published data.

Susceptibility to cefiderocol remained high against *P. aeruginosa* isolates resistant to BLBLI combinations, ranging from 93.6% against ceftazidime-‍avibactam-‍resistant isolates to 98.1% against imipenem-relebactam-‍resistant isolates (Table 2). On the other hand, BLBLI combinations did not show high levels of activity against BLBLI combination-resistant isolates, with the highest susceptibility being 71.8% for ceftolozane-tazobactam against aztreonam-‍avibactam-resistant isolates. Colistin showed 100% susceptibility against BLBLI combination-resistant phenotypes. The activity of antimicrobials against cefiderocol-resistant *P. aeruginosa* was not analyzed due to the low number of isolates (*n* = 10).

Against *P. aeruginosa* isolates that were resistant to both meropenem and c‍e‍f‍t‍a‍z‍i‍d‍i‍m‍e‍-‍a‍v‍i‍b‍a‍c‍t‍a‍m, susceptibility to cefiderocol was 96.7%, which was higher than any BLBLI combination (≤45.0% susceptibility) (Table 2). Similarly, against meropenem- and ceftolozane-tazobactam-resistant isolates, susceptibility to cefiderocol was higher than all tested BLBLI combinations (98.4% vs <55% susceptibility, respectively). Susceptibility rates for antibiotics against antibiotic-resistant *P. aeruginosa* using CLSI breakpoints are reported in Table S2.

In total, 45.3% (227/501) *Acinetobacter* spp. isolates were classified as meropenem resistant (MIC >8 mg/L) (Table 2; see Fig. 1B for MIC distributions). Susceptibility of meropenem-resistant *Acinetobacter* spp. was 85.0% to cefiderocol [European Committee on Antimicrobial Susceptibility Testing (EUCAST) non-‍‍species-‍‍specific pharmacokinetic/pharmacodynamic (PK/PD) breakpoint] and 93.8% to sulbactam-durlobactam (FDA breakpoint) (Table 2) (37, 38). The MIC_90_ was lower for sulbactam-durlobactam (4 mg/L) than cefiderocol (8 mg/L). Cefiderocol susceptibility against s‍u‍l‍b‍ac‍ta‍m‍-‍d‍u‍r‍l‍o‍b‍a‍c‍t‍a‍m‍-‍r‍e‍s‍is‍t‍a‍n‍t‍ isolates (*n* = 15) was 13.3%, while sulbactam-durlobactam susceptibility against cefiderocol-resistant isolates (*n* = 38) was 65.8%. Colistin showed high susceptibility (>92.1%) against all resistant phenotypes. Antimicrobial susceptibility rates in antibiotic-resistant *Acinetobacter* spp. using CLSI or FDA breakpoints are reported in Table S2.

### β-Lactamase genes in meropenem-resistant pathogens

Of the 139 meropenem-resistant *P. aeruginosa* isolates, 2.9% (4/139) were cefiderocol resistant and were analyzed by whole-genome sequencing (WGS), while the remaining isolates were analyzed by PCR for the presence of specific β-lactamase genes. Antimicrobial susceptibility rates in meropenem-resistant *P. aeruginosa* according to β-lactamase genes identified are reported in Table S5A. The most common β-lactamase genes identified in isolates were MBLs [21.6% (30/139)], including 27 isolates with only MBL genes and 3 isolates that each co-harbored 1 KPC, extended-spectrum β-lactamases (ESBL), or acquired OXA gene (Table 3). This trend was similar across all European countries (Table S6A). A small proportion of meropenem-resistant *P. aeruginosa* isolates harbored only ESBL genes [5.8% (8/139)] (Table 3). The most common MBL gene in meropenem-resistant *P. aeruginosa* isolates was VIM [17.3% (24/139), including *bla*_VIM-2_ (15/139) and *bla*_VIM-1_ (7/139)], while 2.9% (4/139) of the isolates harbored IMP (*bla*_IMP-13_: 3/139) and 1.4% (2/139) harbored NDM (*bla*_NDM-1_) genes (Table S6A). Overall, 5.0% (7/139) of isolates harbored GES genes (*bla*_GES-5_: 6/139), 1.4% (2/139) harbored Vietnamese extended-spectrum β-lactamase (VEB) genes (*bla*_VEB-9_), and 0.7% (1/139) harbored *bla*_KPC-2_ or *bla*_OXA-10_ (Table S6A).

**TABLE 3:** Acquired β-lactamase genes identified in meropenem-resistant *P. aeruginosa* isolates (*n* = 139)^*a*^^,^^*b*^

Isolate	β-Lactamase group						Total
	MBL	MBL + KPC	MBL + ESBL	MBL + OXA	ESBL	Negative	
*P. aeruginosa*	27	1	1	1	8	101	139

^*a*^ ESBL, extended-spectrum β-lactamase; KPC, *Klebsiella pneumoniae* carbapenemase; MBL, metallo-β-lactamase; OXA, oxacillinase; PCR, polymerase chain reaction; WGS, whole-genome sequencing.

^*b*^ Data were generated by WGS if meropenem-resistant isolates were resistant to cefiderocol (4/139) or PCR if isolates were susceptible to cefiderocol (135/139). Isolates are grouped as “negative” if only intrinsic β-lactamase genes were present.

Of the 227 meropenem-resistant *Acinetobacter* spp. isolates, 16.7% (38/227) were cefiderocol resistant and were analyzed by WGS, while the remaining isolates were analyzed by PCR for the presence of specific β-lactamase genes. Antimicrobial susceptibility rates in meropenem-‍resistant *Acinetobacter* spp. according to β-lactamase genes identified are reported in Table S5B. The majority of meropenem-‍resistant *Acinetobacter* spp. isolates harbored acquired OXA genes [94.7% (215/227)], including 202 isolates with only acquired OXA genes, 9 that co-‍harbored MBL genes, and 4 that co-harbored ESBL genes (Table 4). This trend was similar across all European countries (Table S6B). A low proportion of isolates harbored only MBL [1.3% (3/227)] genes or co-‍harbored ESBL and KPC genes [0.4% (1/227)] (Table 4). Overall, 85.5% (194/227) of meropenem-resistant *Acinetobacter* spp. isolates harbored *bla*_OXA-23_ or genes of the OXA-23 group, 14.5% (33/227) harbored *bla*_OXA-24_ or genes of the OXA-‍24 group (including 16/227 isolates that co-harbored *bla*_OXA-23-24_), and 3.1% (7/227) harbored *bla*_OXA-72_; 5.3% (12/227) of isolates harbored *bla*_NDM-1_, and 0.4% (1/227) harbored *bla*_KPC-3_ (Table S6B).

**TABLE 4:** Acquired β-lactamase genes identified in meropenem-resistant *Acinetobacter* spp. isolates (*n* = 227)^*a*^^,^^*b*^

Isolate	β-Lactamase group						Total
	OXA	OXA + MBL	OXA + ESBL	MBL	ESBL + KPC	Negative	
*A. baumannii*	156	8	3	3	1	6	177
*A. baumannii* complex	32	–	–	–	–	2	34
*A. bereziniae*	1	–	–	–	–	–	1
*A. nosocomialis*	1	–	–	–	–	–	1
Other *Acinetobacter* spp.	12	1	1	–	–	–	14
Total	202	9	4	3	1	8	227

^*a*^ ESBL, extended-spectrum β-lactamase; KPC, *Klebsiella pneumoniae* carbapenemase; MBL, metallo-β-lactamase; OXA, oxacillinase; PCR, polymerase chain reaction; WGS, whole-genome sequencing.

^*b*^ Data were generated by WGS if meropenem-resistant isolates were resistant to cefiderocol (174/227, including 1 *A. baumannii* complex and 4 other *Acinetobacter* spp.) or PCR if isolates were susceptible to cefiderocol (53/227, including 33 *A. baumannii* complex and 10 other *Acinetobacter* spp.). Isolates are grouped as “negative” if only intrinsic β-lactamase genes were present (*n* = 1) or if no screened β-lactamase gene was present (*n* = 7).

### β-Lactamase genes and other potential resistance mechanisms identified in cefiderocol-resistant pathogens

In total, 10 *P*. *aeruginosa* and 38 *Acinetobacter* spp. (37 *A*. *baumannii*; 1 *Acinetobacter calcoaceticus*) isolates were cefiderocol resistant and were analyzed by WGS.

Four cefiderocol-resistant *P. aeruginosa* isolates were of a sequence type (ST) previously reported (ST235, ST298, ST708, and ST773), while six novel STs were identified (ST4283, ST4290, ST4291, ST4304, ST4305, and ST4306) for isolates collected in France, Germany, Italy, Spain, and the United Kingdom (Table 5). Only one isolate harbored an acquired β-lactamase gene (*bla*_NDM-1_). All cefiderocol-resistant isolates had either mutations in *piuA* and *pirA*-like genes encoding siderophore uptake receptors or these genes were not detected; the majority also had *piuC* (9/10) and *pvdS* (6/10) alterations. Only 20% (2/10) had *ftsl* and *oprD* mutations.

**TABLE 5:** β-Lactamase genes and other potential resistance mechanisms identified in cefiderocol-resistant *P. aeruginosa* isolates (*n* = 10)^*a*^^,^^*b*^

Isolate	Country	FDC MIC (mg/L)	ST	Potential resistance mechanism(s) identified						
				β-Lactamase^*c*^	*ftsl*	*oprD*	*piuA*	*piuC*	*pirA*-like	*pvdS*
1	France	8	4291^*d*^	OXA-396; PDC-543	WT	No gross disruption	Gene not found	Gene not found	A370T	WT
2	France	4	773	OXA-395; PDC-16; NDM-1	WT	No gross disruption	Q34H	R201H	A370T	V180L; H182N
3	France	8	235	OXA-19; OXA-488; PDC-35	WT	No gross disruption	*piuD*	R201H	S20N; T235I; A370T	V180L; H182N
4	Germany	4	298	OXA-848; PDC-219	A244T	Gross disruption	*piuD*	R201H	Gross disruption	V26A
5	Germany	8	4290^*d*^	OXA-1124; PDC-86	WT	No gross disruption	Q34H	T10S; R201H	A13V; A370T	V180L; H182N
6	Italy	8	708	OXA-50; PDC-446	WT	No gross disruption	Q34H	WT	R7H; A370T; Q620K	V180L; T181A; H182N
7	Italy	16	4305^*d*^	OXA-488; PDC-337	WT	No gross disruption	Q34H	R201H	A370T	WT
8	Spain	4	4304^*d*^	OXA-1022; PDC-46	V137M	Gross disruption	Q34H	R201H	Y2S; A370T	WT
9	Spain	4	4306^*d*^	OXA-1188; PDC-5	WT	No gross disruption	*piuD*	V104I	A370T	V180L; T181A; H182N; R186P
10	UK	4	4283^*d*^	OXA-1135; PDC-63	WT	No gross disruption	Gene not found	D49E; E125A; R198Q; V203I	T144S; A208T; A370T; T480A	WT

^*a*^ FDC, cefiderocol; MBL, metallo-β-lactamase; NDM, New Delhi MBL; OXA, oxacillinase; PDC, *Pseudomonas*-derived cephalosporinase; ST, sequence type; UK, United Kingdom; WT, wild type.

^*b*^ Data were generated by whole-genome sequencing. A gene was considered to have a gross disruption if the coding sequence carried a nonsense mutation, frameshift, indels of >20 codons, or ablation of the canonical stop or start codons without a replacement immediately adjacent and in-frame. Genes were listed to be not found if a BLAST search with the reference gene yielded no hit with *E-*value <1*E-*25. Non-β-lactamase genes that were either found to have gross disruptions or mutations or were not found are shown in gray.

^*c*^ Data shown are a curated summary.

^*d*^ Indicates a novel ST.

Half (19/38) of the cefiderocol-resistant *Acinetobacter* spp. isolates were found to be ST2, collected in all six European countries, while the other half consisted of isolates of other STs, including two novel STs from France and Germany (Table 6). A total of 84.2% (32/38) of isolates harbored acquired β-‍‍lactamase genes. Of the ST2 isolates, 73.7% (14/19) harbored *bla*_OXA-23_, 26.3% (5/19) harbored *bla*_OXA-72_, and 5.3% (1/19) harbored *bla*_NDM-1_ or the *bla*_PER-7_ carbapenemase gene. All ST2 isolates had *piuA* mutations and 42.1% (8/19) had *ftsl* mutations, but no *piuC*, *pirA*-like, or *carO* gene mutations were observed. Of the non-‍ST2 isolates, 52.6% (10/19) harbored *bla*_OXA-23_ and 57.9% (11/19) harbored *bla*_NDM-1_. All non-‍ST2 isolates had either *piuA* mutations or this gene was not detected; 57.9% (11/19) had *ftsI* mutations, and 47.4% (9/19) had *pirA*-like gene mutations, or this gene was not detected, but no *carO* mutations were observed.

**TABLE 6:** β-Lactamase genes and other potential resistance mechanisms identified in cefiderocol-resistant *Acinetobacter* spp.^*a*^ isolates (*n* = 38)^*b*^^,^^*c*^

Isolate	Country	FDC MIC (mg/L)	ST	Potential resistance mechanism(s) identified					
				β-Lactamase^*d*^	*ftsI*	*carO*	*piuA*	*piuC*	*pirA*-like
1	Austria	8	2	ADC-74; OXA-66; OXA-72; NDM-1	WT	No gross disruption	G216V; N489D; K658R	WT	WT
2	Austria	4	2	ADC-73; OXA-66; OXA-23	A515V	No gross disruption	Gross disruption	WT	WT
3	France	8	2	ADC-85; OXA-66; OXA-23	A515V	No gross disruption	Gross disruption	WT	WT
4	France	8	2	ADC-73; OXA-66; OXA-23	A515V	No gross disruption	Gross disruption	WT	WT
5	France	4	2	ADC-73; OXA-66; OXA-23	A515V	No gross disruption	Gross disruption	WT	WT
6	France	>32	1	ADC-191; OXA-69; NDM-1; OXA-23	L480I; T511S	No gross disruption	Gross disruption	WT	Gross disruption
7	France	32	1	ADC-191; OXA-69; NDM-1; OXA-23	L480I; T511S	No gross disruption	Gross disruption	WT	Y479N; K543R
8	France	16	85	ADC-165; OXA-94; NDM-1	WT	No gross disruption	Gross disruption (~56% ident to reference)	WT	K475R; H566N
9	France	>32	2163^*e*^	ADC-2; OXA-132	A512T	No gross disruption	I10F; N489D	WT	H566N
10	France	8	1	ADC-204; OXA-69; TEM-Trunc; OXA-23	WT	No gross disruption	L76F; N489D	WT	Y479N; K543R
11	France	32	85	ADC-80; OXA-94; NDM-1	WT	No gross disruption	Gross disruption (~56% ident to reference)	WT	K475R; H566N
12	France	4	203	ADC-Trunc; OXA-65	WT	No gross disruption	S10T; N19D; R42Q; L52I; N58T; A63S; T66Q; H67Q; Q90N; L119V; E125D; I164V; E185D; N206Q; S208E	WT	T340I; H566N
13	Germany	>32	600	ADC-73; OXA-66; TEM-1D; OXA-23	A515V	No gross disruption	Gross disruption	WT	WT
14	Germany	>32	2	ADC-30; OXA-66; PER-7; OXA-23	WT	No gross disruption	G216V; N489D; K658R	WT	WT
15	Germany	16	85	ADC-176; OXA-94; NDM-1	WT	No gross disruption	Gross disruption (and ~56% ident to reference)	WT	K475R; H566N
16	Germany	4	636	ADC-74; OXA-66; OXA-72	WT	No gross disruption	Gross disruption	WT	WT
17	Germany	>32	2262^*e*^	ADC-11; OXA-66; OXA-72; PER-1	WT	No gross disruption	G216V; N489D; K658R	WT	WT
18	Italy	16	2	ADC-33; OXA-82; OXA-23	H370Y	Gross disruption	G216V; N489D; K658R	WT	WT
19	Italy	4	2	ADC-73; OXA-66; OXA-23	A515V	No gross disruption	G216V; N489D; K658R	WT	WT
20	Italy	4	2	ADC-33; OXA-82; OXA-23	WT	No gross disruption	G216V; N489D; K658R	WT	WT
21	Italy	16	2	ADC-33; OXA-82; OXA-23	WT	No gross disruption	G216V; N489D; K658R	WT	WT
22	Italy	4	2	ADC-33; OXA-82; OXA-23	WT	No gross disruption	G216V; N489D; K658R	WT	WT
23	Italy	>32	2	ADC-30; OXA-66; OXA-72	WT	No gross disruption	G216V; N489D; K658R	WT	WT
24	Italy	16	2	ADC-30; OXA-66; OXA-72	WT	No gross disruption	G216V; N489D; K658R	WT	WT
25	Italy	>32	2	ADC-30; OXA-66; OXA-72	WT	No gross disruption	G216V; N489D; K658R	WT	WT
26	Italy	>32	2	ADC-30; OXA-66; OXA-72	WT	No gross disruption	G216V; N489D; K658R	WT	WT
27	Italy	16	600	ADC-73; OXA-66; TEM-1D; NDM-1; OXA-23	A515V	No gross disruption	Gross disruption	WT	WT
28	Italy	16	600	ADC-73; OXA-66; TEM-1D; NDM-1; OXA-23	A515V	No gross disruption	Gross disruption	WT	WT
29	Italy	16	600	ADC-73; OXA-66; TEM-1D; NDM-1; OXA-23	A515V	No gross disruption	Gross disruption	WT	WT
30	Italy	8	600	ADC-73; OXA-66; TEM-1D; NDM-1; OXA-23	A515V	No gross disruption	Gross disruption	WT	WT
31	Italy	8	600	ADC-Trunc; OXA-66; TEM-1D; NDM-1; OXA-23	A515V	No gross disruption	Gross disruption	WT	WT
32	Italy	16	600	ADC-73; OXA-66; TEM-1D; NDM-1; OXA-23	A515V	No gross disruption	Gross disruption	WT	WT
33	Italy	4	2	ADC-33; OXA-82; OXA-23	WT	No gross disruption	G216V; N489D; K658R	WT	WT
34	Italy	4	2	ADC-33; OXA-82; OXA-23	WT	No gross disruption	G216V; N489D; K658R	WT	WT
35	Spain	4	79	ADC-1; OXA-65	WT	No gross disruption	Gene not found	Gene not found	WT
36	Spain	>32	2	ADC-30; OXA-66; OXA-23	K235N	No gross disruption	G216V; N489D; K658R	WT	WT
37^*f*^	UK	8	432	ADC-295; OXA-1189	V72I; E115A; T179M; V343I; A345S; Q405E; A435V; E471Q; A483P; P604S; E605V	No gross disruption	Gross disruption	Gene not found	Gene not found
38	UK	>32	2	ADC-73; OXA-66; TEM-1D; OXA-23	A515V	No gross disruption	G216V; N489D; K658R	WT	WT

^*a*^ Isolates shown are *A. baumannii* unless otherwise indicated.

^*b*^ ADC, *Acinetobacter*-derived cephalosporinase; FDC, cefiderocol; MBL, metallo-β-lactamase; NDM, New Delhi MBL; OXA, oxacillinase; ST, sequence type; Trunc, truncated; UK, United Kingdom; WT, wild type.

^*c*^ Data were generated by whole-genome sequencing. A gene was considered to have a gross disruption if the coding sequence carried a nonsense mutation, frameshift, indels of >20 codons, or ablation of the canonical stop or start codons without a replacement immediately adjacent and in-frame. Genes were listed to be not found if a BLAST search with the reference gene yielded no hit with *E-*value <1*E-*25. Non-β-lactamase genes that were either found to have gross disruptions or mutations or were not found are shown in gray.

^*d*^ Data shown are a curated summary.

^*e*^ Indicates a novel ST.

^*f*^ *A. calcoaceticus*.

## DISCUSSION

This study provides additional data on the *in vitro* susceptibilities of cefiderocol and BLBLI combinations, including those still in development, against a large collection of European isolates of glucose non-fermenting Gram-negative bacteria. The data collected in this study are from a greater number of sites per European country compared with the longitudinal SENTRY and SIDERO surveillance programs, which are more geographically spread (35, 36).

The susceptibility rate for cefiderocol was higher than BLBLI combinations (including those still in development, such as aztreonam-avibactam and cefepime-taniborbactam) against *P. aeruginosa* overall (98.9% vs 83.3%–91.4%, respectively) and meropenem-resistant isolates (97.8% vs ≤59.7%). These observations are consistent with similar previous *in vitro* studies (5, 26, 39–45). It is important to note that meropenem-resistant *P. aeruginosa* isolates in this study were defined according to the EUCAST MIC resistance breakpoint for high-dose (2 g), extended (3-h)-infusion meropenem (>8 mg/L), to represent isolates that are meropenem resistant even when treated with the highest meropenem dose available to patients. As some previous studies have defined meropenem-‍resistant/non-susceptible *P. ‍aeruginosa* according to the EUCAST MIC resistance breakpoint for standard-dose meropenem (>2 mg/L), susceptibility rates for BLBLI ‍combinations tested in previous studies may be higher (39, 43).

Of the β-lactamase genes observed in meropenem-resistant *P. aeruginosa* isolates, most were MBLs (most commonly VIM), against which cefiderocol retains high activity, in contrast to most BLBLI combinations (46). Low frequencies (≤5%) of IMP, NDM (*bla*_NDM-1_), and GES were observed, lower than previously published data (6). The 71.2% of meropenem-resistant *P. aeruginosa* isolates which did not harbor β-lactamase genes of interest likely exhibited non-β-lactamase mechanisms of resistance, such as increased expression of efflux systems, chromosomal cephalosporinase activity, or reduced porin expression (3–6, 47).

Susceptibility to cefiderocol was higher than to BLBLI combinations against BLBLI combination-resistant *P. aeruginosa* (93.6%–98.1% vs 12.2%–71.8%, respectively). Similarly, susceptibility to cefiderocol was high against meropenem-‍resistant *P. aeruginosa* resistant to ceftazidime-avibactam or ceftolozane-tazobactam (≥96.7%), while ceftolozane-tazobactam and ceftazidime-‍avibactam both had poorer activity (≤30.6%). This is indicative of a low degree of cross resistance between cefiderocol and BLBLI combinations and a particularly high degree of cross resistance between ceftolozane-tazobactam and ceftazidime-avibactam. Previous studies have also shown cefiderocol to have much higher activity than ceftazidime-avibactam or ceftolozane-tazobactam against ceftazidime-avibactam- or ceftolozane-tazobactam-resistant *P. aeruginosa* (6, 48, 49). Given that 11%–23% of *P. aeruginosa* isolates show resistance to ceftazidime-avibactam or ceftolozane-tazobactam, cefiderocol would be the preferred agent over BLBLI combinations for treatment of infections caused by such isolates. Even aztreonam-avibactam and cefepime-taniborbactam, which are still in development, showed lower susceptibility (≤54.8%) compared with cefiderocol (≥96.7%) against meropenem-resistant *P. aeruginosa* resistant to ceftazidime-‍avibactam or ceftolozane-tazobactam. Lower activity of cefepime-‍taniborbactam was previously demonstrated against ceftolozane-tazobactam- and ceftazidime-avibactam-resistant/-non-susceptible *P. aeruginosa*, as taniborbactam does not fully restore cefepime activity in some BLBLI combination-resistant *P. aeruginosa* isolates, such as IMP producers (Table S5) (39, 50). Further, studies on the *in vitro* activity of aztreonam-avibactam showed poor activity against *P. aeruginosa* overall (44, 45).

Cefiderocol-resistant *P. aeruginosa* isolates were collected across Europe in this study. Although isolates were not collected under a surveillance program, the diverse range of STs indicates that cefiderocol resistance in *P. ‍aeruginosa* arises from specific clones and is not due to clonal expansion. Although there were no clear patterns of resistance mechanisms in cefiderocol-resistant *P. aeruginosa* isolates in this study, previous observations have noted the common presence of mutations in genes encoding the PiuA and PirA receptors required for the uptake of siderophore conjugates (51–54) and the role of mutations in the *ftsI* gene encoding PBP3 (50). Further investigations are required to confirm whether mutations in *piuA*, *pirA,* and *ftsI* impact drug resistance or are natural polymorphisms.

*Acinetobacter* spp. are particularly difficult to treat due to the prevalence of antimicrobial resistance (2). However, both cefiderocol and sulbactam-‍durlobactam demonstrated good *in vitro* activity against *Acinetobacter* spp. overall (>90% susceptibility) and meropenem-resistant isolates (85.0% and 93.8% susceptibility, respectively), in agreement with previous studies (5, 55, 56). Against *Acinetobacter* ‍spp. overall, cefiderocol demonstrated a lower MIC_90_ than sulbactam-‍durlobactam. To the authors’ knowledge, this is the first published study directly comparing the *in vitro* activity of cefiderocol and sulbactam-‍durlobactam, which was recently approved by the United States Food and Drug Administration for the treatment of hospital-acquired/ventilator-associated bacterial pneumonia caused by susceptible *A. baumannii* complex in adults (57, 58). While susceptibility to sulbactam-durlobactam was higher than to cefiderocol against meropenem-resistant *Acinetobacter* spp., the FDA breakpoint for sulbactam-durlobactam (≤4 mg/L) was used, as EUCAST breakpoints are not available (37). Had the CLSI breakpoint been used in place of the EUCAST non-‍species-specific PK/PD breakpoint for cefiderocol (≤4 mg/L vs ≤2 mg/L), cefiderocol susceptibility against meropenem-resistant *Acinetobacter* spp. would be more comparable to sulbactam-durlobactam (88.5%) (Table S2) (59).

The majority of these meropenem-resistant *Acinetobacter* spp. isolates harbored *bla*_OXA-23_ (85.5%), which has been recognized as the most prevalent carbapenem-hydrolyzing class D β-lactamase in CRAB isolates (60–62), although there were low numbers of isolates with MBL genes; 5.3% harbored *bla*_NDM-1_ and none harbored *bla*_VIM_.

Cefiderocol-resistant *Acinetobacter* spp. accounted for 7.6% of *Acinetobacter* ‍spp. collected in this study, against which sulbactam-durlobactam demonstrated good *in vitro* activity. Although some cefiderocol-resistant isolates had similar genotypic and phenotypic data, which may suggest clonality, isolates were not collected under a surveillance program and numbers were low. Mutations in *piuA* were found in 97.4% of isolates, suggesting that *piuA* may be the major iron-‍regulated outer membrane protein involved in the uptake of cefiderocol in *Acinetobacter* spp. Half of all cefiderocol-resistant *Acinetobacter* spp. isolates had mutations in the *ftsI* gene encoding PBP3, which cefiderocol is known to inhibit (52). The retained activity of sulbactam-durlobactam against cefiderocol-resistant *Acinetobacter* spp. suggests that there does not appear to be any cross resistance due to PBP3 target-site mutations. This was unexpected, as sulbactam also inhibits PBP3, and sulbactam-durlobactam resistance has previously been attributed to PBP3 mutations (32, 63); in addition, *Acinetobacter* spp. resistant to sulbactam-‍durlobactam (3.0% of isolates) also had low susceptibility to cefiderocol (13.3%). It may be a concern that sulbactam-durlobactam resistance is being observed in Europe this early in the use of this treatment, possibly as a consequence of prior exposure to ampicillin-sulbactam. However, this study was not designed to comprehensively investigate mechanisms of resistance in non-fermenter isolates.

The data of this study do provide insights into the therapeutic options for infections due to *P. aeruginosa* and *Acinetobacter* spp. with resistant phenotypes. The low levels of cross resistance observed between cefiderocol and any BLBLI combination, with the exception of sulbactam-durlobactam, support the concept that cefiderocol should be tested at the same time as these BLBLI combinations to allow clinicians to choose effective treatment(s) for non-fermenter infections from all available options. It is particularly important that all effective treatment(s) are identified and considered, as current treatment options are limited (33, 34). Importantly, the high cross resistance observed between ceftazidime-avibactam and ceftolozane-tazobactam in this and other studies suggests that cycling between these treatments to treat infections due to *P. aeruginosa* is unlikely to be an appropriate option (64–66). The susceptibilities of aztreonam-‍avibactam and cefepime-‍‍taniborbactam against *P. aeruginosa* resistant to meropenem and both meropenem and BLBLI combinations were low, which also suggests that these will not be good treatment options for infections due to *P. aeruginosa*.

Although susceptibility to colistin was high against meropenem-resistant *P. aeruginosa* and *Acinetobacter* spp. isolates, colistin is known to have concerningly high rates of nephrotoxicity and poor tissue penetration, particularly in the lungs (67–69), and the majority of non-fermenter isolates in this study were from respiratory tract infections. Colistin is also not recommended by EUCAST for monotherapy and is not associated with a clinical monotherapy breakpoint (70).

There are several limitations to this study. Isolates were only collected from six European countries, and up to 35 non-fermenter isolates per participating site were included. Hence, there were low numbers of some isolates resistant to at least one antimicrobial, particularly cefiderocol. Ceftazidime-avibactam was tested using a validated commercial method, while other antimicrobials were tested using custom plates. A selection of potential mechanisms of resistance was only screened for in meropenem- and cefiderocol-‍resistant isolates, using different methodologies and screening panels, and excluding analysis of expression levels of genes or other potential mechanisms of BLBLI combination resistance, such as PBP1 and PBP2. Therefore, robust interpretations of resistance mechanisms and any cross resistance could not be made. The STs of cefiderocol-‍susceptible isolates were not identified, so clonal expansion of isolates with resistant phenotypes was not determined; however, these non-surveillance data would not have accurately reflected clonal epidemiology in Europe. Lastly, *in vitro* data cannot replace clinical studies in patients, and *in ‍vitro* activity may not reflect *in vivo* efficacy of a therapy in clinical practice.

### Conclusions

These results confirm the high levels of *in vitro* activity of cefiderocol against Gram-‍‍negative *P. aeruginosa* and *Acinetobacter* spp. isolates from Europe, including both meropenem-resistant isolates and those resistant to recent BLBLI combinations commonly used in first-‍line treatment of CR infections. Cefiderocol often had high *in ‍vitro* activity where the majority of BLBLI combinations did not, and there was no apparent cross resistance between cefiderocol and BLBLI combinations, with the exception of sulbactam-‍durlobactam.

## MATERIALS AND METHODS

### Clinical isolates

Between 01 January and 31 December 2020, Gram-negative clinical isolates from hospitalized inpatients were collected at 49 sites across Austria, France, Germany, Italy, Spain, and the United Kingdom (see Table S7 for details of participating centers). Each site was requested to collect 20 *P*. *aeruginosa* and 15 *A*. *baumannii* (Enterobacterales isolates were also collected as part of the overall study, for which the methods and results are reported elsewhere). Isolates included those from all infection sources, with the exception of the urinary tract. Only one isolate of the same genus and species was allowed per patient. Matrix-assisted laser desorption/ionization-time-of-flight mass spectrometry was used for species identification at International Health Management Associates (IHMA) Europe Sàrl (Monthey, Switzerland).

### Antimicrobial susceptibility testing

Antimicrobial susceptibility testing was performed on all collected isolates at IHMA Europe Sàrl. Isolates were stored at −70°C before testing by broth microdilution for the determination of MICs. Antimicrobials tested were cefiderocol, meropenem, ceftazidime-avibactam, ceftolozane-tazobactam, meropenem-vaborbactam, imipenem-relebactam, aztreonam-avibactam, cefepime-taniborbactam, and colistin against *P. aeruginosa*, and cefiderocol, meropenem, sulbactam-durlobactam, and colistin against *Acinetobacter* spp. (see Table S8 for suppliers of agents).

International Organization for Standardization 20776-1 susceptibility testing standards and EUCAST guidance were followed for the preparation of antimicrobials for testing and MIC determinations (71, 72); tryptic soy agar plates containing 5% sheep blood were sourced from Liofilchem (Roseto degli Abruzzi, Italy; product code: 11037), cation-adjusted Mueller-Hinton broth was sourced from Becton Dickinson (Franklin Lakes, NJ, USA; product code: 212322), and iron-depleted cation-adjusted Mueller-Hinton broth (used for cefiderocol testing) was prepared by IHMA Europe Sàrl. This excludes ceftazidime-avibactam, for which MIC values were only available when Sensititre freeze-dried panels (Thermo Fisher Scientific Inc., Waltham, MA, USA) were used in the preparation of ceftazidime-avibactam for testing. All antibiotics were tested daily using the quality control strains *P. aeruginosa* ATCC 27853 (as recommended by EUCAST and CLSI, and in line with guidance from CLSI) and *A. baumannii* ATCC 13304 (in line with guidance from CLSI, where a same-species quality control strain was not recommended by EUCAST) (59, 73). The MIC values for each tested antibiotic were manually read as the lowest concentration inhibiting visible growth. For cefiderocol and meropenem, MIC values were determined more than once; a third MIC determination was carried out if MIC values differed by >1 dilution, and the geometric mean was reported.

### Analysis

Antimicrobial susceptibility results were interpreted in accordance with EUCAST clinical breakpoints (v.14.0, 2024) (37) (see Table S9 for breakpoints used).

Meropenem resistance was defined using a breakpoint of MIC >8 mg/L, relating to high-dose, extended-infusion (2 g, 3-h infusion) meropenem; similarly, isolates with a meropenem MIC >8 mg/L when tested with a fixed vaborbactam concentration of 8 mg/L were considered resistant to meropenem-vaborbactam. Aztreonam-avibactam, cefepime-taniborbactam, and sulbactam-durlobactam do not currently have approved EUCAST MIC breakpoints; nor does cefiderocol for *Acinetobacter* spp. For aztreonam-avibactam and cefepime-taniborbactam, EUCAST breakpoints for high-dose aztreonam and high-dose cefepime alone were used. For *Acinetobacter* spp., the non-species-specific PK/PD breakpoint (37) was used for cefiderocol and the FDA breakpoint (38) was used for sulbactam-durlobactam. For colistin, EUCAST ECOFF values were used.

### Identification of β-lactamase genes in meropenem-resistant isolates

Isolates with a meropenem MIC >8 mg/L and a cefiderocol MIC ≤2 mg/L were analyzed by PCR (performed by IHMA Europe Sàrl) to identify the presence of β-lactamase genes that may confer meropenem resistance (see Table S10 for genes and primers used). Data on β-lactamase genes in isolates that were meropenem resistant (meropenem MIC >8 mg/L) and cefiderocol resistant (MIC >2 mg/L) were generated by WGS (see below).

DNA extraction was performed from a single colony obtained from a fresh tryptic soy blood agar culture for each isolate, using the QIAGEN TissueLyser II instrument (Hilden, Germany) as per manufacturer instructions. Preparations then underwent PCR amplification and sequencing to screen for the presence of genes encoding clinically relevant β-lactamases: ESBLs (*bla*_SHV_, *bla*_TEM_, *bla*_CTX-M_, *bla*_VEB_, *bla*_PER_, *bla*_GES_), AmpCs [*bla*_ACC_, *bla*_CMY I/MOX_, *bla*_CMY II_, *bla*_DHA_, *bla*_FOX_, *bla*_ACT-MIR_, *bla*_PDC_ (in *P. aeruginosa* only)], and carbapenemases (*bla*_KPC_, *bla*_OXA_, *bla*_NDM_, *bla*_IMP_, *bla*_VIM_, *bla*_SPM_, *bla*_GIM_, *bla*_GES_). Amplicons were sequenced by Fasteris (Geneva, Switzerland) and then analyzed using SeqScape Software 3 (Thermo Fisher Scientific Inc.; Waltham, MA, USA). Limited sequencing was used to screen *bla*_TEM_ and *bla*_SHV_ to identify TEM-type and SHV-type enzymes containing amino acid substitutions common to ESBLs (*bla*_TEM_: amino acid 104, 164, 238, 240; *bla*_SHV_: amino acid 146, 179, 238, 240) and to screen *bla*_CTX-M_ (groups 1, 2, 8, 9, and 25) to identify CTX-M-type enzymes containing the D240G amino acid substitution associated with elevated ceftazidime MIC values. Genes encoding SHV-type and TEM-type enzymes were reported as ESBL or original-spectrum β-lactamase genes. The 16S ribosomal DNA for all isolates was also amplified by PCR and sequenced for bacterial identification.

### Identification of β-lactamase genes and other potential resistance mechanisms in cefiderocol-resistant isolates

Isolates with a cefiderocol MIC >2 mg/L were analyzed by WGS to identify possible mechanisms of resistance. DNA isolation was performed using the QIAGEN QIAamp DNA Mini kit, and library preparation was performed using the Illumina DNA Prep kit (San Diego, CA, USA) at IHMA, Inc. (Schaumburg, IL, USA). Libraries were then shipped to Azenta (South Plainfield, NJ, USA), where short-read WGS (2 × 150 base pairs; paired-end) was performed on an Illumina HiSeq platform to a 100× depth of coverage. Quality control was performed using the CheckM lineage workflow (74–76) to assure low contamination (≤5%) and completeness of assemblies (≥‍95%) were achieved. The multilocus sequence typing scheme Pasteur was used to determine relatedness of *Acinetobacter* spp. isolates.

Genomic assemblies were created using the QIAGEN CLC Genomics workbench (v.21.0.5). In order to identify β-lactamase genes of interest, assemblies were queried using the ResFinder database (77) with coverage and identity thresholds of ≥35% and ≥72%, respectively. Genes identified with <100% identity or coverage were evaluated for a variant by pairwise alignment to a reference sequence using the ResFinder database (77). Variants were defined using the Bacterial Antimicrobial Resistance Reference Gene Database from the National Center for Biotechnology Information (Bioproject 313047).

Non-β-lactamase genes of interest in this study included those encoding PBP3 (*ftsI*), porins [*oprD* (*P. aeruginosa*) and *carO* (*Acinetobacter* spp.)], and those related to iron acquisition [*pirA*-like, *piuA*, *piuC,* and *pvdS* (*P. aeruginosa*)]. Genes were analyzed by pairwise alignment and classified as wild type if they had 100% amino acid sequence identity to the species-specific reference sequence (Table S11). These genes were also screened for gross disruption vs species-specific reference sequences (Table S11) and were considered to have gross disruption if the coding sequence carried a nonsense mutation, frameshift, indels of >20 codons, or ablation of the canonical start or stop codons without a replacement immediately adjacent and in-frame. Genes were not considered disrupted if there were ablated start or stop codons immediately adjacent to intact, in-frame start or stop codons. Genes were listed to be not found if a BLAST search with the reference gene yielded no hit with *E*-‍value <1*E*−25.

## Data Availability

Whole-genome sequencing variants were defined using the Bacterial Antimicrobial Resistance Reference Gene Database from the National Center for Biotechnology Information (Bioproject number: PRJNA313047). Data are available from Shionogi B.V. upon reasonable request.

## SUPPLEMENTAL MATERIAL

Supplemental material spectrum.03836-23-s0001.docx Figures S1; Tables S1 to S11.

ASM does not own the copyrights to Supplemental Material that may be linked to, or accessed through, an article. The authors have granted ASM a non-exclusive, world-wide license to publish the Supplemental Material files. Please contact the corresponding author directly for reuse.
