# Supplementary material for: In vitro activity of cefiderocol against European Pseudomonas aeruginosa and Acinetobacter spp., including isolates resistant to meropenem and recent β-lactam/β-lactamase inhibitor combinations
Source: Microbiol Spectr. 2024 Mar 14;12(4):e03836-23. doi: 10.1128/spectrum.03836-23 (PMC10986614; doi:10.1128/spectrum.03836-23)
Supplement: Supplemental material — Figures S1; Tables S1 to S11. [file spectrum.03836-23-s0001.docx]

## SUPPLEMENTARY MATERIALS

| 1. *Pseudomonas aeruginosa* (n = 950)   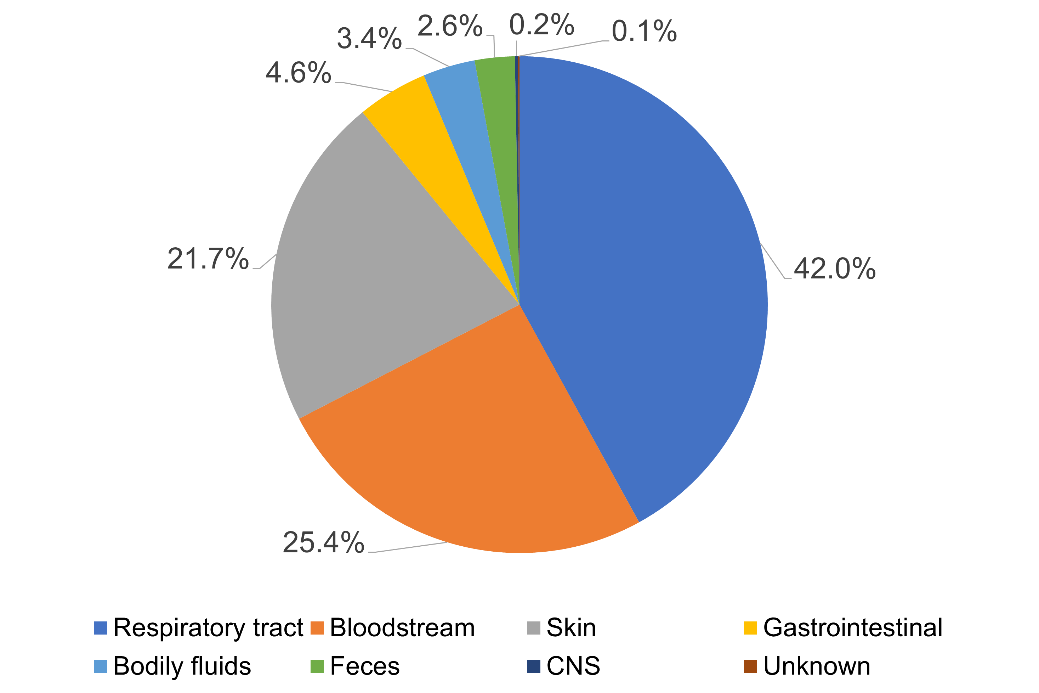 | 1. *Acinetobacter* spp. (n = 501)*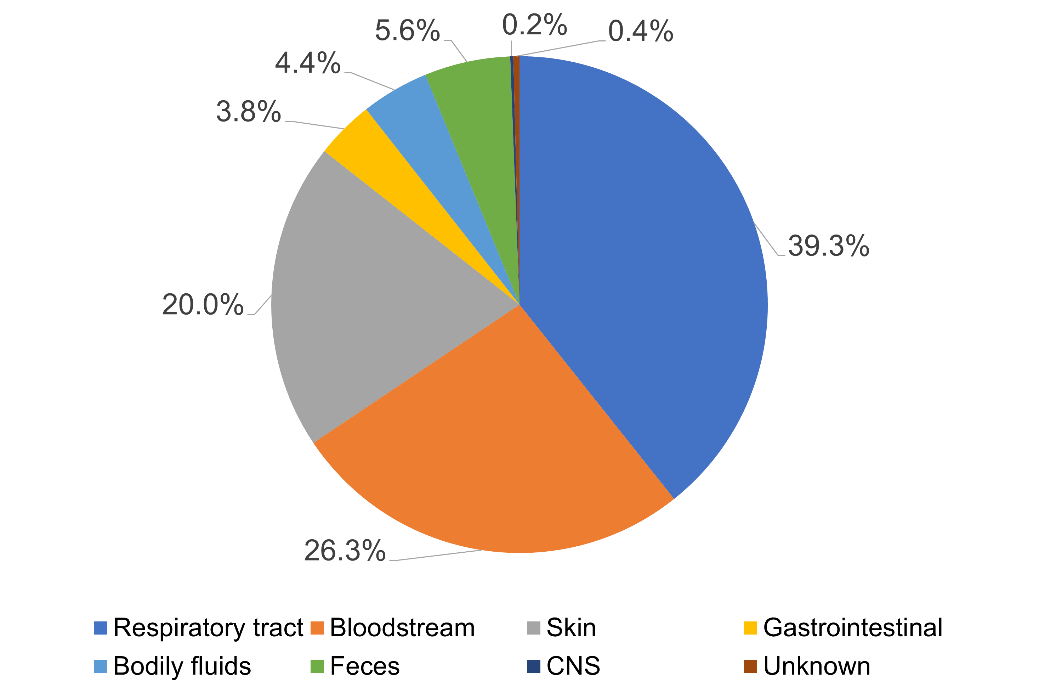* |
| --- | --- |
|  |  |

**FIG S1** *P. aeruginosa* and *Acinetobacter* spp. isolates by infection source.

CNS, central nervous system.

**TABLE S1** *P. aeruginosa* and *Acinetobacter* spp. isolates by country

| **Isolate** | **France** | **Germany/Austria** | **Italy** | **Spain** | **UK** |
| --- | --- | --- | --- | --- | --- |
| *P. aeruginosa*^a^ | 226 (29.4) | 161 (28.2) | 206 (26.7) | 212 (28.0) | 145 (29.4) |
| *Acinetobacter* spp.^a^ | 113 (14.7) | 76 (13.3) | 150 (19.5) | 102 (13.5) | 60 (12.2) |
| *A. baumannii* complex | 105 (13.7) | 72 (12.6) | 138 (17.9) | 92 (12.2) | 51 (10.3) |
| *A. baylyi* | 1 (0.1) | 0 | 0 | 0 | 1 (0.2) |
| *A. bereziniae* | 0 | 0 | 0 | 1 (0.1) | 0 |
| *A. dijkshoorniae* | 1 (0.1) | 1 (0.2) | 0 | 3 (0.4) | 1 (0.2) |
| *A. johnsonii* | 0 | 0 | 1 (0.1) | 1 (0.1) | 0 |
| *Acinetobacter* (unspeciated) | 6 (0.8) | 3 (0.5) | 11 (1.4) | 5 (0.7) | 7 (1.4) |

^a^ Percentages shown are from the number of collected isolates in each country.

Data are shown as n (%).

**TABLE S2** *In vitro* activity of cefiderocol, BLBLI combinations and other relevant antibiotics against *P. aeruginosa* and *Acinetobacter*spp. isolates, including those with resistant phenotypes, according to CLSI breakpoints

|  | **FDC** | | **MEM** | | **CZA** | | **C/T** | | **MVB** | | **I-R** | | **ATM-AVI** | | **FEP-TAN** | | **SUL-DUR** | | **(CST)** | |
| --- | --- | --- | --- | --- | --- | --- | --- | --- | --- | --- | --- | --- | --- | --- | --- | --- | --- | --- | --- | --- |
| **Isolates (n)** | **MIC_90_ (mg/L)** | **S (%)** | **MIC_90_ (mg/L)** | **S  (%)** | **MIC_90_ (mg/L)** | **S (%)** | **MIC_90_ (mg/L)** | **S (%)** | **MIC_90_ (mg/L)** | **S  (%)** | **MIC_90_ (mg/L)** | **S (%)** | **MIC_90_ (mg/L)** | **S  (%)** | **MIC_90_ (mg/L)** | **S  (%)** | **MIC_90_ (mg/L)** | **S (%)** | **MIC_90_ (mg/L)** | **I (%)** |
| *P. aeruginosa*  (950) | 1 | 99.5 | 16 | 69.2 | 8 | 90.1 | 8 | 89.1 | 16 | 71.6 | 4 | 83.3 | 32 | 71.1 | 8 | 91.4 | N/A | N/A | (1) | (99.7) |
| MEM-R (139) | 1 | 100 |  |  | > 16 | 56.8 | > 32 | 55.4 | > 32 | 0.0 | > 16 | 12.2 | > 32 | 12.2 | > 32 | 59.7 | N/A | N/A | (1) | (100) |
| CZA-R (94) | 2 | 95.7 | > 16 | 22.3 |  |  | > 32 | 36.2 | > 32 | 23.4 | > 16 | 37.2 | > 32 | 28.7 | > 32 | 55.3 | N/A | N/A | (1) | (100) |
| C/T-R (104) | 2 | 95.2 | > 16 | 15.4 | > 16 | 42.3 |  |  | > 32 | 21.2 | > 16 | 36.5 | 32 | 27.9 | > 32 | 59.6 | N/A | N/A | (1) | (100) |
| MVB-R (122) | 1 | 100 | > 16 | 0.0 | > 16 | 53.3 | > 32 | 53.3 |  |  | > 16 | 10.7 | > 32 | 13.1 | > 32 | 59.0 | N/A | N/A | (1) | (100) |
| I-R-R (159) | 1 | 100 | > 16 | 2.5 | > 16 | 62.9 | > 32 | 58.5 | > 32 | 4.4 |  |  | > 32 | 26.4 | 32 | 64.8 | N/A | N/A | (1) | (100) |
| ATM-AVI-R (131) | 2 | 98.5 | > 16 | 24.4 | > 16 | 67.2 | > 32 | 71.8 | > 32 | 26.0 | > 16 | 44.3 |  |  | 32 | 61.8 | N/A | N/A | (1) | (99.2) |
| FEP-TAN-R (82) | 2 | 98.8 | > 16 | 14.6 | > 16 | 48.8 | > 32 | 48.8 | > 32 | 15.9 | > 16 | 31.7 | > 32 | 20.7 |  |  | N/A | N/A | (1) | (100) |
| MEM-R and CZA-R (60) | 2 | 100 |  |  |  |  | > 32 | 28.3 | > 32 | 0.0 | > 16 | 8.3 | > 32 | 18.3 | > 32 | 43.3 | N/A | N/A | (1) | (100) |
| MEM-R and  C/T-R (62) | 2 | 100 |  |  | > 16 | 30.6 |  |  | > 32 | 0.0 | > 16 | 9.7 | 32 | 21.0 | > 32 | 54.8 | N/A | N/A | (1) | (100) |
| *Acinetobacter* spp*.* (501)^a^ | 2 | 94.4 | > 16 | 52.9 | N/A | N/A | N/A | N/A | N/A | N/A | N/A | N/A | N/A | N/A | N/A | N/A | 4 | 97.0 | (0.5) | (98.4) |
| FDC-R (38) | > 32 | 26.3 | > 16 | 7.9 | N/A | N/A | N/A | N/A | N/A | N/A | N/A | N/A | N/A | N/A | N/A | N/A | > 16 | 65.8 | (2) | (92.1) |
| MEM-R (227) | 8 | 88.5 | > 16 | 0.0 | N/A | N/A | N/A | N/A | N/A | N/A | N/A | N/A | N/A | N/A | N/A | N/A | 4 | 93.8 | (0.5) | (97.4) |
| SUL-DUR-R (15) | > 32 | 13.3 | > 16 | 0.0 | N/A | N/A | N/A | N/A | N/A | N/A | N/A | N/A | N/A | N/A | N/A | N/A | > 16 | 0.0 | (≤ 0.25) | (100) |

^a^ Includes 458 *A. baumannii* complex isolates.
ATM-AVI, aztreonam-avibactam; BLBLI, β-lactam/β-lactamase inhibitor; CLSI, Clinical and Laboratory Standards Institute; CST, colistin; C/T, ceftolozane/tazobactam; CZA, ceftazidime-avibactam; EUCAST, European Committee on Antimicrobial Susceptibility Testing; FDA, Food and Drug Administration; FDC, cefiderocol; FEP-TAN, cefepime-taniborbactam; I, intermediate; I-R, imipenem-‍relebactam; MEM, meropenem; MVB, meropenem-vaborbactam; N/A, not applicable; R, resistant; S, susceptibility; SUL-‍DUR, sulbactam-durlobactam.

Antibiotics were tested against *P. aeruginosa* and/or *Acinetobacter* spp. based on expected use in a real-world setting. Resistant phenotypes were defined according to EUCAST breakpoints (v.14.0, 2024). Susceptibility was assessed according to CLSI breakpoints (2023), except for sulbactam-durlobactam where FDA breakpoints were used. Colistin data are presented using the CLSI intermediate breakpoint and are shown in parentheses as colistin does not have a CLSI susceptibility breakpoint and is not recommended for monotherapy (as per EUCAST v.14.0 2024 and CLSI 2023 guidance).

**TABLE S3** *In vitro* activity of cefiderocol, BLBLI combinations, and other relevant antibiotics against *P. aeruginosa* and *Acinetobacter* spp. isolates, by country

1. France

| **Isolates (n)** | **FDC** | | **MEM** | | **ATM-AVI** | | **C/T** | | **CZA** | | **MVB** | | **I-R** | | **FEP-TAN** | | **SUL-DUR** | | **(CST)** | |
| --- | --- | --- | --- | --- | --- | --- | --- | --- | --- | --- | --- | --- | --- | --- | --- | --- | --- | --- | --- | --- |
|  | **MIC_90_ (mg/L)** | **S (%)** | **MIC_90_ (mg/L)** | **S/I (%)** | **MIC_90_ (mg/L)** | **S (%)** | **MIC_90_ (mg/L)** | **S (%)** | **MIC_90_ (mg/L)** | **S (%)** | **MIC_90_ (mg/L)** | **S/I (%)** | **MIC_90_ (mg/L)** | **S (%)** | **MIC_90_ (mg/L)** | **S/I (%)** | **MIC_90_ (mg/L)** | **S (%)** | **MIC_90_ (mg/L)** | **S (%)** |
| *P. aeruginosa*  (226) | 1 | 98.7 | 16 | 87.2 | 32 | 87.2 | 8 | 87.2 | 8 | 90.3 | 16 | 87.6 | 4 | 87.2 | 8 | 92.0 | N/A | N/A | (1) | (100) |
| *Acinetobacter* spp*.* (113)^a^ | 2 | 91.2 | > 16 | 79.6 | N/A | N/A | N/A | N/A | N/A | N/A | N/A | N/A | N/A | N/A | N/A | N/A | 2 | 95.6 | (0.5) | (100) |

1. Germany/Austria

| **Isolates (n)** | **FDC** | | **MEM** | | **ATM-AVI** | | **C/T** | | **CZA** | | **MVB** | | **I-R** | | **FEP-TAN** | | **SUL-DUR** | | **(CST)** | |
| --- | --- | --- | --- | --- | --- | --- | --- | --- | --- | --- | --- | --- | --- | --- | --- | --- | --- | --- | --- | --- |
|  | **MIC_90_ (mg/L)** | **S (%)** | **MIC_90_ (mg/L)** | **S/I (%)** | **MIC_90_ (mg/L)** | **S (%)** | **MIC_90_ (mg/L)** | **S (%)** | **MIC_90_ (mg/L)** | **S (%)** | **MIC_90_ (mg/L)** | **S/I (%)** | **MIC_90_ (mg/L)** | **S (%)** | **MIC_90_ (mg/L)** | **S/I (%)** | **MIC_90_ (mg/L)** | **S (%)** | **MIC_90_ (mg/L)** | **S (%)** |
| *P. aeruginosa*  (161) | 0.5 | 98.8 | 16 | 88.8 | 32 | 89.4 | 4 | 93.8 | 8 | 95.7 | 8 | 91.9 | 4 | 83.2 | 8 | 90.7 | N/A | N/A | (1) | (98.1) |
| *Acinetobacter* spp*.* (76)^a^ | 2 | 90.8 | > 16 | 81.6 | N/A | N/A | N/A | N/A | N/A | N/A | N/A | N/A | N/A | N/A | N/A | N/A | 2 | 96.1 | (0.5) | (100) |

1. Italy

| **Isolates (n)** | **FDC** | | **MEM** | | **ATM-AVI** | | **C/T** | | **CZA** | | **MVB** | | **I-R** | | **FEP-TAN** | | **SUL-DUR** | | **(CST)** | |
| --- | --- | --- | --- | --- | --- | --- | --- | --- | --- | --- | --- | --- | --- | --- | --- | --- | --- | --- | --- | --- |
|  | **MIC_90_ (mg/L)** | **S (%)** | **MIC_90_ (mg/L)** | **S/I (%)** | **MIC_90_ (mg/L)** | **S (%)** | **MIC_90_ (mg/L)** | **S (%)** | **MIC_90_ (mg/L)** | **S (%)** | **MIC_90_ (mg/L)** | **S/I (%)** | **MIC_90_ (mg/L)** | **S (%)** | **MIC_90_ (mg/L)** | **S/I (%)** | **MIC_90_ (mg/L)** | **S (%)** | **MIC_90_ (mg/L)** | **S (%)** |
| *P. aeruginosa*  (206) | 1 | 99.0 | > 16 | 77.2 | 32 | 81.6 | > 32 | 80.1 | >16 | 81.1 | > 32 | 78.6 | > 16 | 73.3 | 16 | 88.3 | N/A | N/A | (1) | (100) |
| *Acinetobacter* spp*.* (150)^a^ | 4 | 88.7 | > 16 | 10.0 | N/A | N/A | N/A | N/A | N/A | N/A | N/A | N/A | N/A | N/A | N/A | N/A | 4 | 95.3 | (0.5) | (96.7) |

1. Spain

| **Isolates (n)** | **FDC** | | **MEM** | | **ATM-AVI** | | **C/T** | | **CZA** | | **MVB** | | **I-R** | | **FEP-TAN** | | **SUL-DUR** | | **(CST)** | |
| --- | --- | --- | --- | --- | --- | --- | --- | --- | --- | --- | --- | --- | --- | --- | --- | --- | --- | --- | --- | --- |
|  | **MIC_90_ (mg/L)** | **S (%)** | **MIC_90_ (mg/L)** | **S/I (%)** | **MIC_90_ (mg/L)** | **S (%)** | **MIC_90_ (mg/L)** | **S (%)** | **MIC_90_ (mg/L)** | **S (%)** | **MIC_90_ (mg/L)** | **S/I (%)** | **MIC_90_ (mg/L)** | **S (%)** | **MIC_90_ (mg/L)** | **S/I (%)** | **MIC_90_ (mg/L)** | **S (%)** | **MIC_90_ (mg/L)** | **S (%)** |
| *P. aeruginosa*  (212) | 1 | 99.1 | 16 | 87.3 | 32 | 85.4 | 4 | 92.9 | 8 | 90.1 | 8 | 90.1 | 4 | 86.3 | 8 | 91.5 | N/A | N/A | (1) | (100) |
| *Acinetobacter* spp*.* (102)^a^ | 1 | 98.0 | > 16 | 50.0 | N/A | N/A | N/A | N/A | N/A | N/A | N/A | N/A | N/A | N/A | N/A | N/A | 2 | 100 | (0.5) | (99.0) |

1. UK

| **Isolates (n)** | **FDC** | | **MEM** | | **ATM-AVI** | | **C/T** | | **CZA** | | **MVB** | | **I-R** | | **FEP-TAN** | | **SUL-DUR** | | **(CST)** | |
| --- | --- | --- | --- | --- | --- | --- | --- | --- | --- | --- | --- | --- | --- | --- | --- | --- | --- | --- | --- | --- |
|  | **MIC_90_ (mg/L)** | **S (%)** | **MIC_90_ (mg/L)** | **S/I (%)** | **MIC_90_ (mg/L)** | **S (%)** | **MIC_90_ (mg/L)** | **S (%)** | **MIC_90_ (mg/L)** | **S (%)** | **MIC_90_ (mg/L)** | **S/I (%)** | **MIC_90_ (mg/L)** | **S (%)** | **MIC_90_ (mg/L)** | **S/I (%)** | **MIC_90_ (mg/L)** | **S (%)** | **MIC_90_ (mg/L)** | **S (%)** |
| *P. aeruginosa*  (145) | 1 | 99.3 | 16 | 87.6 | 32 | 89.0 | 4 | 93.8 | 4 | 96.6 | 16 | 89.0 | 4 | 86.9 | 8 | 95.2 | N/A | N/A | (1) | (100) |
| *Acinetobacter* spp*.* (60)^a^ | 1 | 96.7 | 1 | 93.3 | N/A | N/A | N/A | N/A | N/A | N/A | N/A | N/A | N/A | N/A | N/A | N/A | 2 | 100 | (0.5) | (96.7) |

^a^ Data include *A. baumannii* complex isolates (France, n = 105; Germany/Austria, n = 72; Italy, n = 138; Spain, n = 92; UK, n = 51).

ATM-AVI, aztreonam-avibactam; BLBLI, β-lactam/β-lactamase inhibitor; CST, colistin; C/T, ceftolozane/tazobactam; CZA, ceftazidime-avibactam; ECOFF, epidemiological cut-off; EUCAST, European Committee on Antimicrobial Susceptibility Testing; FDA, Food and Drug Administration; FDC, cefiderocol; FEP-TAN, cefepime-taniborbactam; I, susceptible, increased exposure; I‑R, imipenem-relebactam; MEM, meropenem; MVB, meropenem-vaborbactam; N/A, not applicable; S, susceptibility.

Antimicrobials were tested against *P. aeruginosa* and/or *Acinetobacter* spp. based on expected use in a real-world setting. Susceptibility was assessed according EUCAST breakpoints (including non-species-specific PK/PD breakpoints, high dosage breakpoints, and breakpoints for the agent without inhibitor, where applicable), except for sulbactam-durlobactam and colistin where FDA breakpoints and ECOFF values were used, respectively. Data on susceptibility to colistin are colored grey as colistin is not recommended for monotherapy and is not associated with a clinical monotherapy breakpoint (as per EUCAST v.14.0 guidance).

**TABLE S4** *In vitro* activity of cefiderocol, BLBLI combinations and other relevant antibiotics against *P. aeruginosa* and *Acinetobacter* spp. isolates with resistance phenotypes, by country

1. France

| **Isolates** | **n** | **Susceptibility^a^, %** | | | | | | | | | | | | | | | | | |  |
| --- | --- | --- | --- | --- | --- | --- | --- | --- | --- | --- | --- | --- | --- | --- | --- | --- | --- | --- | --- | --- |
|  |  | **FDC** | | | | | | **MEM** | | **CZA** | | **C/T** | | **MVB** | | **I-R** | **ATM-AVI** | **FEP-TAN** | **SUL-DUR** | **(CST)** |
| ***P. aeruginosa*** | **226** |  | | | | | | | | | | | | | | | | | |  |
| MEM-R | 29 | 96.6 | | | | | |  | | 55.2 | | 58.6 | | 3.4 | | 17.2 | 34.5 | 55.2 | N/A | (100) |
| CZA-R | 22 | 90.9 | | | | | | 40.9 | |  | | 18.2 | | 40.9 | | 45.5 | 31.8 | 40.9 | N/A | (100) |
| C/T-R | 29 | 89.7 | | | | | | 58.6 | | 37.9 | |  | | 58.6 | | 55.2 | 51.7 | 55.2 | N/A | (100) |
| MVB-R | 28 | 96.4 | | | | | | 0 | | 53.6 | | 57.1 | |  | | 17.9 | 32.1 | 53.6 | N/A | (100) |
| I-R-R | 29 | 96.6 | | | | | | 17.2 | | 58.6 | | 55.2 | | 20.7 | |  | 41.4 | 55.2 | N/A | (100) |
| ATM-AVI-R | 29 | 96.6 | | | | | | 34.5 | | 48.3 | | 51.7 | | 34.5 | | 41.4 |  | 58.6 | N/A | (100) |
| ***Acinetobacter* spp.** | **113** |  |  |  |  |  |  | |  | |  | |  | |  |  |  |  |  |  |
| MEM-R | 23 | 65.2 | | | | | |  | | N/A | | N/A | | N/A | | N/A | N/A | N/A | 82.6 | (100) |

1. Germany/Austria

| **Isolates** | **n** | **Susceptibility^a^, %** | | | | | | | | |  |  |  |  |  |  |  |  |
| --- | --- | --- | --- | --- | --- | --- | --- | --- | --- | --- | --- | --- | --- | --- | --- | --- | --- | --- |
|  |  | **FDC** | **MEM** | **CZA** | **C/T** | **MVB** | **I-R** | **ATM-AVI** | **FEP-TAN** | **SUL-DUR** | **(CST)** |  |  |  |  |  |  |  |
| ***P. aeruginosa*** | **161** |  | | | | | | | | |  |  |  |  |  |  |  |  |
| I-R-R | 27 | 100 | 37.0 | 77.8 | 74.1 | 55.6 |  | 66.7 | 59.3 | N/A | (100) |  |  |  |  |  |  |  |
| ***Acinetobacter* spp.** | **76** |  | | | | | | | | |  |  |  |  |  |  |  | N/A |

1. Italy

| **Isolates** | **n** | **Susceptibility^a^, %** | | | | | | | | |  |
| --- | --- | --- | --- | --- | --- | --- | --- | --- | --- | --- | --- |
|  |  | **FDC** | **MEM** | **CZA** | **C/T** | **MVB** | **I-R** | **ATM-AVI** | **FEP-TAN** | **SUL-DUR** | **(CST)** |
| ***P. aeruginosa*** | **206** |  | | | | | | | | |  |
| MEM-R | 47 | 100 |  | 42.6 | 44.7 | 6.4 | 6.4 | 42.6 | 59.6 | N/A | (100) |
| CZA-R | 39 | 94.9 | 30.8 |  | 38.5 | 35.9 | 30.8 | 64.1 | 64.1 | N/A | (100) |
| C/T-R | 41 | 95.1 | 36.6 | 41.5 |  | 39.0 | 26.8 | 65.9 | 68.3 | N/A | (100) |
| MVB-R | 44 | 100 | 0 | 43.2 | 43.2 |  | 4.5 | 43.2 | 61.4 | N/A | (100) |
| I-R-R | 55 | 100 | 20.0 | 50.9 | 45.5 | 23.6 |  | 56.4 | 70.9 | N/A | (100) |
| ATM-AVI-R | 38 | 97.4 | 28.9 | 63.2 | 63.2 | 34.2 | 36.8 |  | 57.9 | N/A | (100) |
| FEP-TAN-R | 24 | 100 | 20.8 | 41.7 | 45.8 | 29.2 | 33.3 | 33.3 |  | N/A | (100) |
| ***Acinetobacter* spp.** | **150** |  |  |  |  |  |  |  |  |  |  |
| MEM-R | 135 | 87.4 |  | N/A | N/A | N/A | N/A | N/A | N/A | 94.8 | (97.0) |

1. Spain

| **Isolates** | **n** | **Susceptibility^a^, %** | | | | | | | | |  |  |  |  |  |  |
| --- | --- | --- | --- | --- | --- | --- | --- | --- | --- | --- | --- | --- | --- | --- | --- | --- |
|  |  | **FDC** | **MEM** | **CZA** | **C/T** | **MVB** | **I-R** | **ATM-AVI** | **FEP-TAN** | **SUL-DUR** | **(CST)** |  |  |  |  |  |
| ***P. aeruginosa*** | **212** |  | | | | | | | | |  |  |  |  |  |  |
| MEM-R | 27 | 92.6 |  | 66.7 | 63.0 | 22.2 | 18.5 | 51.9 | 59.3 | N/A | (100) |  |  |  |  |  |
| CZA-R | 21 | 95.2 | 57.1 |  | 57.1 | 61.9 | 57.1 | 66.7 | 57.1 | N/A | (100) |  |  |  |  |  |
| MVB-R | 21 | 90.5 | 0 | 61.9 | 57.1 |  | 14.3 | 47.6 | 52.4 | N/A | (100) |  |  |  |  |  |
| I-R-R | 29 | 93.1 | 24.1 | 69.0 | 69.0 | 37.9 |  | 58.6 | 62.1 | N/A | (100) |  |  |  |  |  |
| ATM-AVI-R | 31 | 93.5 | 58.1 | 77.4 | 90.3 | 64.5 | 61.3 |  | 61.3 | N/A | (100) |  |  |  |  |  |
| ***Acinetobacter* spp.** | **102** |  | | | | | | | | |  |  |  |  |  | N/A |
| MEM-R | 51 | 98.0 |  | N/A | N/A | N/A | N/A | N/A | N/A | 100 | (98.0) |  |  |  |  |  |

^a^ Refers to susceptibility, or susceptibility with increased exposure for meropenem, meropenem-vaborbactam, aztreonam‑avibactam, and cefepime-taniborbactam.

ATM-AVI, aztreonam-avibactam; BLBLI, β-lactam/β-lactamase inhibitor; CST, colistin; C/T, ceftolozane/tazobactam; CZA, ceftazidime-avibactam; ECOFF, epidemiological cut-off; EUCAST, European Committee on Antimicrobial Susceptibility Testing; FDA, Food and Drug Administration; FDC, cefiderocol; FEP-TAN, cefepime‑taniborbactam; I-R, imipenem-relebactam; MEM, meropenem; MVB, meropenem-vaborbactam; N/A, not applicable; R, resistant.

Antimicrobials were tested against *P. aeruginosa* and/or *Acinetobacter* spp. based on expected use in a real-world setting. Results are not reported for isolates tested against antibiotics to which they had an expected resistance phenotype. Susceptibility was assessed according EUCAST breakpoints (including non-species-specific PK/PD breakpoints, high dosage breakpoints, and breakpoints for the agent without inhibitor, where applicable), except for sulbactam-durlobactam and colistin where FDA breakpoints and ECOFF values were used, respectively. Data are shown where n≥20 isolates were available; numbers of isolates with resistant phenotypes from the UK were < 20. Data on susceptibility to colistin are shown in parentheses in grey as colistin is not recommended for monotherapy and is not associated with a clinical monotherapy breakpoint (as per EUCAST v.14.0 guidance).

**Table S5** *In vitro* activity of cefiderocol and BLBLI combinations against meropenem-resistant *P. aeruginosa* and *Acinetobacter*spp. isolates, according to relevant acquired β-lactamase genes identified

1. Meropenem-resistant *P. aeruginosa*^a^

| **Isolates (n) producing:** | **FDC** | | | **CZA** | | | **C/T** | | | **MVB** | | | **I-R** | | | **ATM-AVI** | | | **FEP-TAN** | | |
| --- | --- | --- | --- | --- | --- | --- | --- | --- | --- | --- | --- | --- | --- | --- | --- | --- | --- | --- | --- | --- | --- |
|  | **MIC_90_ (mg/L)** | **S (%)** | | **MIC_90_ (mg/L)** | **S (%)** | | **MIC_90_ (mg/L)** | **S (%)** | | **MIC_90_ (mg/L)** | **S (%)** | | **MIC_90_ (mg/L)** | **S (%)** | | **MIC_90_ (mg/L)** | **S (%)** | | **MIC_90_ (mg/L)** | **S (%)** | |
|  |  | **EUCAST** | **CLSI** |  | **EUCAST** | **CLSI** |  | **EUCAST** | **CLSI** |  | **EUCAST** | **CLSI** |  | **EUCAST** | **CLSI** |  | **EUCAST** | **CLSI** |  | **EUCAST** | **CLSI** |
| NDM (2) | 4 | 50.0 | 100 | 16 | 0 | 0 | 32 | 0 | 0 | 32 | 0 | 0 | 16 | 0 | 0 | 32 | 50.0 | 50.0 | 32 | 0 | 0 |
| VIM (24) | 1 | 100 | 100 | 16 | 0 | 0 | 32 | 0 | 0 | 32 | 0 | 0 | 16 | 0 | 0 | 16 | 79.2 | 33.3 | > 32 | 62.5 | 62.5 |
| IMP (4) | 0.25 | 100 | 100 | 16 | 0 | 0 | 32 | 0 | 0 | 32 | 0 | 0 | 16 | 0 | 0 | 32 | 50.0 | 0 | 32 | 25.0 | 25.0 |
| KPC (1) | 2 | 100 | 100 | 16 | 0 | 0 | 32 | 0 | 0 | 32 | 0 | 0 | 16 | 0 | 0 | 32 | 0 | 0 | 8 | 100 | 100 |
| OXA (4) | 4 | 25.0 | 100 | > 16 | 25.0 | 25.0 | > 32 | 50.0 | 50.0 | 32 | 0 | 0 | > 16 | 0 | 0 | 32 | 50.0 | 50.0 | 32 | 0 | 0 |
| VEB (2) | 2 | 100 | 100 | > 16 | 0 | 0 | 32 | 0 | 0 | 32 | 0 | 0 | 16 | 50.0 | 50.0 | 32 | 0 | 0 | 32 | 0 | 0 |
| GES (7) | 0.5 | 100 | 100 | 8 | 57.1 | 57.1 | 16 | 0 | 0 | 32 | 0 | 0 | > 16 | 0 | 0 | 32 | 28.6 | 28.6 | 16 | 57.1 | 57.1 |

1. Meropenem-resistant *Acinetobacter* spp.^b^

| **Isolates (n) producing:** | **FDC** | | | **SUL-DUR** | | |
| --- | --- | --- | --- | --- | --- | --- |
|  | **MIC_90_ (mg/L)** | **S (%)** | | **MIC_90_ (mg/L)** | **S (%)** | |
|  |  | **EUCAST** | **CLSI** |  | **EUCAST^c^** | **FDA** |
| OXA (219) | 8 | 84.5 | 88.1 | 4 | N/A | 93.6 |
| OXA-23GR (193) | 4 | 87.5 | 91.2 | 4 | N/A | 94.8 |
| OXA-24GR (32) | 2 | 90.6 | 93.8 | 4 | N/A | 100 |
| NDM (12) | 32 | 0 | 0 | 16 | N/A | 0 |
| KPC (1) | 2 | 100 | 100 | 0.5 | N/A | 100 |
| TEM (61)^d^ | 8 | 86.9 | 86.9 | 8 | N/A | 86.9 |
| PER (2) | 32 | 0 | 0 | 1 | N/A | 100 |

^a^ Of the 139 meropenem-resistant *P. aeruginosa* isolates, 38 harbored at least one acquired β-lactamase gene.

^b^ Of the 227 meropenem-resistant *Acinetobacter* spp. isolates, 218 harbored at least one acquired β-lactamase gene.

^c^ No published EUCAST (v.14.0) breakpoint.

^d^ Excludes one truncated TEM gene deemed to be non-functional.

ATM-AVI, aztreonam-avibactam; BLBLI, β-lactam/β-lactamase inhibitor; CLSI, Clinical and Laboratory Standards Institute; CST, colistin; C/T, ceftolozane/tazobactam; CZA, ceftazidime-avibactam; EUCAST, European Committee on Antimicrobial Susceptibility Testing; FDA, Food and Drug Administration; FEP-TAN, cefepime-taniborbactam; GES, Guiana extended-spectrum β-lactamase; GR, group; I, intermediate; IMP, imipenemase; I-R, imipenem-relebactam; KPC, *K. pneumoniae* carbapenemase; MBL, metallo-‍β-‍lactamase; MEM, meropenem; N/A, not applicable; NDM, New Delhi MBL; OXA, oxacillinase; PER, *Pseudomonas* extended resistant; S, susceptibility; SUL-DUR, sulbactam-durlobactam; VEB, Vietnamese extended-spectrum β-lactamase; VIM, Verona integron-borne MBL.

Antibiotics were tested against *P. aeruginosa* and/or *Acinetobacter* spp. based on expected use in a real-world setting. Resistant phenotypes were defined according to EUCAST breakpoints (v.14.0, 2024). Susceptibility was assessed according to EUCAST (v.14.0, 2024) and CLSI (2023) breakpoints, with the exception of sulbactam-durlobactam, which was assessed according to FDA breakpoints.

**Table S6** MIC values and β-lactamase genes identified in meropenem-resistant *P. aeruginosa* and *Acinetobacter* spp. isolates

1. Meropenem-resistant *P. aeruginosa* isolates (n = 139)

| **Country** | **MIC (mg/L)** | | | | | | | | | **β-Lactamase genes identified** |
| --- | --- | --- | --- | --- | --- | --- | --- | --- | --- | --- |
|  | **FDC** | **MEM** | **CZA** | **C/T** | **MVB** | **I-R** | **ATM-AVI** | **FEP-TAN** | **(CST)** |  |
| Austria | 0.06 | > 16 | > 16 | > 32 | > 32 | > 16 | 16 | 8 | (1) | VIM-2 |
| Austria | 0.06 | > 16 | 16 | 2 | > 32 | 16 | > 32 | 16 | (0.5) | - |
| Austria | 0.25 | > 16 | 16 | 2 | 32 | 4 | > 32 | 16 | (0.5) | - |
| Austria | 0.5 | > 16 | 8 | 2 | 32 | 4 | 32 | 16 | (1) | - |
| Austria | 0.5 | 16 | 16 | 4 | 16 | 16 | 32 | 8 | (1) | - |
| Austria | 0.12 | 16 | 4 | 1 | 16 | 2 | 32 | 8 | (0.5) | - |
| Austria | 0.5 | > 16 | > 16 | > 32 | > 32 | > 16 | 16 | > 32 | (0.5) | VIM-1 |
| France | 0.12 | 16 | 4 | 1 | 16 | 4 | 32 | 8 | (1) | - |
| France | 0.5 | 16 | > 16 | 8 | 16 | 8 | > 32 | 16 | (1) | - |
| France | 0.06 | 16 | 4 | 4 | 16 | 8 | 8 | 2 | (1) | PDC-Type |
| France | 0.5 | 16 | > 16 | 32 | 32 | 16 | > 32 | 32 | (0.5) | - |
| France | 2 | > 16 | > 16 | > 32 | > 32 | > 16 | 32 | 8 | (0.5) | KPC-2; VIM-2 |
| France | 0.12 | 16 | 8 | 2 | 16 | 4 | 16 | 16 | (0.5) | PDC-Type |
| France | 0.06 | 16 | 4 | 1 | 16 | 4 | 32 | 4 | (1) | - |
| France | 0.5 | > 16 | > 16 | > 32 | 32 | 2 | > 32 | > 32 | (1) | - |
| France | 0.5 | > 16 | > 16 | 8 | 32 | 8 | 32 | 16 | (1) | - |
| France | 0.12 | > 16 | 16 | 4 | > 32 | 4 | > 32 | 16 | (1) | - |
| France | 0.25 | > 16 | 8 | 4 | 32 | 2 | 16 | 8 | (1) | - |
| France | 0.06 | 16 | 8 | 2 | 16 | 2 | 32 | 8 | (1) | - |
| France | 0.12 | > 16 | 8 | 4 | 32 | 16 | 32 | 16 | (1) | PDC-19A |
| France | < 0.03 | 16 | 4 | 2 | 16 | 4 | 16 | 8 | (1) | - |
| France | < 0.03 | 16 | 4 | 2 | 8 | 4 | 16 | 8 | (1) | - |
| France | 0.5 | 16 | 2 | 1 | 16 | 1 | 16 | 4 | (0.5) | - |
| France | 1 | > 16 | 4 | 2 | 16 | 4 | > 32 | 8 | (1) | - |
| France | 1 | 16 | 8 | 2 | 16 | 16 | 32 | 8 | (0.5) | - |
| France | 0.06 | > 16 | 4 | 4 | 32 | > 16 | 32 | 16 | (1) | - |
| France | 2 | > 16 | > 16 | > 32 | > 32 | > 16 | 32 | > 32 | (1) | IMP-13 |
| France | 1 | > 16 | 8 | 2 | 16 | 4 | 32 | 8 | (1) | - |
| France | 0.12 | 16 | 4 | 1 | 16 | 4 | 32 | 8 | (1) | - |
| France | 2 | > 16 | > 16 | > 32 | 32 | 8 | > 32 | 32 | (1) | - |
| France | 0.25 | > 16 | > 16 | > 32 | > 32 | > 16 | 16 | > 32 | (0.5) | IMP-13 |
| France^a^ | 4 | > 16 | > 16 | > 32 | > 32 | > 16 | 4 | > 32 | (0.5) | NDM-1; OXA‑395; PDC-16 |
| France | 2 | 16 | 16 | > 32 | 16 | 2 | 32 | 8 | (0.5) | VEB-9 |
| France | 1 | > 16 | > 16 | > 32 | > 32 | > 16 | 8 | 32 | (1) | VIM-1 |
| France | 0.5 | 16 | 4 | 2 | 16 | 16 | 32 | 8 | (1) | - |
| France | 0.12 | > 16 | > 16 | > 32 | > 32 | > 16 | 8 | 8 | (1) | VIM-2 |
| Germany | 0.5 | 16 | 4 | > 32 | 8 | 16 | 32 | > 32 | (0.5) | - |
| Germany | < 0.03 | 16 | 4 | 4 | 8 | 16 | < 1 | 32 | (1) | - |
| Germany | 0.12 | 16 | 8 | 2 | 16 | 4 | > 32 | 16 | (0.5) | PDC-34 |
| Germany | 0.12 | 16 | 8 | 1 | 16 | 4 | 32 | 8 | (1) | - |
| Germany | 0.06 | 16 | 8 | 2 | 8 | 16 | 16 | 4 | (1) | - |
| Germany | < 0.03 | 16 | 8 | 8 | 8 | 8 | 16 | 4 | (< 0.25) | PDC-5 |
| Germany | 0.25 | > 16 | 8 | 2 | 32 | 8 | > 32 | 16 | (1) | - |
| Germany | 0.03 | 16 | 8 | 16 | 16 | 16 | 16 | 8 | (< 0.25) | PDC-Type |
| Germany | < 0.03 | 16 | 16 | 8 | 16 | 16 | 8 | 8 | (0.5) | - |
| Germany | 0.25 | 16 | 4 | 1 | 8 | 4 | 16 | 8 | (1) | - |
| Germany | 0.5 | 16 | 8 | 1 | 16 | 4 | 32 | 8 | (1) | - |
| Italy | 0.25 | > 16 | > 16 | > 32 | > 32 | > 16 | 16 | 4 | (1) | VIM-2 |
| Italy | 0.5 | 16 | 16 | 4 | 16 | 4 | 32 | 8 | (1) | - |
| Italy | 0.06 | 16 | > 16 | 2 | 16 | 8 | 16 | 4 | (1) | - |
| Italy | 0.25 | > 16 | > 16 | > 32 | > 32 | > 16 | 16 | 8 | (1) | VIM-2 |
| Italy | 0.5 | > 16 | > 16 | > 32 | > 32 | > 16 | 32 | > 32 | (1) | VIM-1 |
| Italy | 0.25 | > 16 | > 16 | > 32 | > 32 | > 16 | 32 | 4 | (1) | VIM-2 |
| Italy | 0.25 | > 16 | > 16 | > 32 | > 32 | > 16 | 16 | 4 | (1) | VIM-2 |
| Italy | 0.25 | > 16 | > 16 | > 32 | > 32 | > 16 | 16 | 4 | (1) | VIM-2 |
| Italy | 0.06 | > 16 | 4 | 4 | 16 | 4 | 16 | 16 | (1) | PDC-Type |
| Italy | 0.25 | 16 | 8 | 16 | 16 | 4 | 32 | 8 | (1) | - |
| Italy | 0.25 | 16 | 8 | 1 | 16 | 4 | 32 | 8 | (1) | - |
| Italy | 0.06 | > 16 | > 16 | > 32 | > 32 | > 16 | 16 | > 32 | (1) | VIM-2 |
| Italy | 0.25 | > 16 | > 16 | > 32 | > 32 | > 16 | 16 | 8 | (1) | VIM-2 |
| Italy | 0.5 | > 16 | > 16 | > 32 | > 32 | > 16 | 8 | 8 | (0.5) | VIM-28 |
| Italy | 0.06 | 16 | > 16 | > 32 | 8 | 2 | 32 | 16 | (0.5) | PDC-325 |
| Italy | 0.06 | 16 | 4 | 2 | 16 | 4 | 8 | 8 | (0.5) | PDC-35 |
| Italy | 2 | 16 | 8 | 2 | 16 | 4 | 32 | 8 | (1) | - |
| Italy | 0.12 | > 16 | > 16 | > 32 | > 32 | > 16 | 16 | 8 | (1) | VIM-2 |
| Italy | 0.25 | > 16 | > 16 | > 32 | > 32 | > 16 | 16 | 8 | (1) | VIM-2 |
| Italy | 0.25 | 16 | > 16 | 2 | 8 | 16 | 16 | 16 | (0.5) | - |
| Italy | 0.25 | > 16 | > 16 | > 32 | > 32 | > 16 | 32 | 16 | (1) | VIM-2 |
| Italy | 0.5 | > 16 | > 16 | > 32 | > 32 | > 16 | 16 | 8 | (1) | VIM-2 |
| Italy | 0.25 | 16 | > 16 | 2 | 16 | 4 | > 32 | 8 | (1) | - |
| Italy | 0.25 | 16 | 16 | 2 | 32 | 2 | > 32 | 32 | (0.5) | - |
| Italy | 0.12 | 16 | 16 | 4 | 16 | 8 | 32 | 16 | (1) | - |
| Italy | 0.25 | > 16 | 4 | 16 | > 32 | > 16 | 32 | 8 | (0.5) | GES-Type |
| Italy | 0.25 | > 16 | 8 | 16 | > 32 | > 16 | 32 | 16 | (0.5) | GES-5 |
| Italy | 0.25 | > 16 | 4 | 16 | > 32 | > 16 | 32 | 16 | (1) | GES-5 |
| Italy | 0.25 | 16 | 1 | 8 | 16 | 4 | 8 | 4 | (0.5) | - |
| Italy | 0.5 | > 16 | > 16 | > 32 | > 32 | > 16 | 8 | > 32 | (1) | VIM-1 |
| Italy | 0.25 | > 16 | 16 | 4 | 32 | 4 | > 32 | 16 | (1) | - |
| Italy | 0.5 | 16 | > 16 | 4 | 16 | 4 | 8 | 4 | (0.5) | - |
| Italy | 0.25 | > 16 | 8 | 16 | > 32 | > 16 | 32 | 16 | (0.5) | GES-5 |
| Italy | 0.25 | > 16 | 8 | 16 | > 32 | > 16 | 32 | 8 | (0.5) | GES-5 |
| Italy | 0.06 | 16 | 4 | 8 | 16 | 4 | 32 | 16 | (0.5) | PDC-Type |
| Italy | 0.25 | > 16 | 16 | 4 | 16 | 4 | > 32 | 8 | (1) | - |
| Italy | 0.12 | 16 | 4 | 2 | 16 | 4 | 16 | 8 | (1) | - |
| Italy | 0.25 | 16 | 8 | 2 | 16 | 4 | > 32 | 8 | (0.5) | - |
| Italy | 0.25 | 16 | 8 | 1 | 16 | 4 | 32 | 8 | (0.5) | - |
| Italy | 0.06 | 16 | 8 | 2 | 16 | 1 | 32 | 16 | (1) | PDC-19A |
| Italy | 0.5 | 16 | 4 | 1 | 8 | 4 | 32 | 4 | (1) | PDC-117 |
| Italy | < 0.03 | > 16 | 16 | 2 | > 32 | 16 | > 32 | 16 | (0.5) | - |
| Italy | 0.25 | 16 | 8 | 8 | 16 | 8 | 16 | 8 | (1) | IMP-33 |
| Italy | < 0.03 | > 16 | > 16 | > 32 | > 32 | > 16 | 32 | > 32 | (1) | VIM-1 |
| Italy | 0.12 | > 16 | 8 | 2 | 32 | 4 | > 32 | 16 | (0.5) | - |
| Italy | 0.06 | 16 | 4 | 1 | 16 | 4 | 32 | 8 | (1) | - |
| Italy | 0.5 | > 16 | > 16 | > 32 | > 32 | > 16 | 8 | > 32 | (0.5) | VIM-1 |
| Spain | 0.5 | 16 | 8 | 16 | 8 | 2 | 16 | 4 | (1) | - |
| Spain | 0.25 | 16 | 8 | 4 | 8 | 2 | 16 | 4 | (0.5) | - |
| Spain | 0.06 | > 16 | 8 | 2 | > 32 | 2 | > 32 | 8 | (0.5) | - |
| Spain | 0.25 | 16 | 4 | 2 | 16 | 4 | 16 | 8 | (1) | PDC-Type |
| Spain | 0.12 | 16 | 16 | 2 | 16 | 16 | 32 | 8 | (< 0.25) | PDC-3 |
| Spain | 0.25 | 16 | > 16 | > 32 | 16 | 2 | 16 | 16 | (0.5) | PDC-Type |
| Spain | 0.06 | 16 | 8 | 2 | 8 | 4 | 16 | 8 | (0.5) | PDC-19A |
| Spain | 0.25 | 16 | 16 | 8 | 16 | 16 | 16 | 16 | (1) | - |
| Spain | 0.5 | > 16 | 4 | 16 | > 32 | 16 | 8 | 8 | (1) | GES-5 |
| Spain | 0.25 | > 16 | 4 | 16 | > 32 | > 16 | 8 | 8 | (1) | GES-5 |
| Spain | 0.25 | 16 | 4 | 2 | 8 | 4 | 32 | 16 | (0.5) | PDC-Type |
| Spain | 0.5 | > 16 | 8 | 2 | 16 | 16 | 32 | 32 | (0.5) | - |
| Spain | 0.25 | 16 | > 16 | 4 | 8 | 8 | 32 | 4 | (0.5) | PDC-5 |
| Spain | 0.5 | 16 | 8 | 1 | 16 | 16 | 16 | 8 | (0.5) | - |
| Spain | 0.25 | > 16 | 4 | 1 | > 32 | 4 | 16 | 4 | (1) | - |
| Spain^a^ | 2 | > 16 | > 16 | > 32 | 16 | 16 | 8 | 16 | (0.5) | OXA-10; OXA-396; PDC-8; VIM-1 |
| Spain | 1 | > 16 | > 16 | > 32 | > 32 | > 16 | 8 | 8 | (2) | VIM-2 |
| Spain | 1 | 16 | 4 | 8 | 16 | 4 | 16 | 4 | (1) | PDC-Type |
| Spain | 0.5 | 16 | > 16 | > 32 | 16 | 4 | 32 | 16 | (0.5) | - |
| Spain | 0.12 | 16 | 4 | 2 | 8 | 4 | 16 | 8 | (1) | PDC-Type |
| Spain^a^ | 4 | > 16 | 8 | 4 | 16 | 8 | 32 | 8 | (1) | OXA-1022; PDC-46 |
| Spain | 0.25 | 16 | 8 | 2 | 16 | 4 | 32 | 8 | (2) | - |
| Spain | < 0.03 | 16 | 8 | 4 | 16 | 4 | 32 | 16 | (0.5) | PDC-Type |
| Spain | 0.25 | > 16 | 8 | 2 | 16 | 2 | 32 | 16 | (2) | PDC-31 |
| Spain | 0.12 | > 16 | > 16 | > 32 | > 32 | > 16 | 32 | > 32 | (1) | IMP-13 |
| Spain^a^ | 4 | 16 | 16 | 4 | 16 | 4 | 32 | 16 | (0.5) | OXA-50-Type; PDC-5 |
| Spain | 0.12 | 16 | 4 | 2 | 16 | 8 | 32 | 32 | (1) | - |
| UK | 0.25 | > 16 | > 16 | > 32 | > 32 | 16 | 32 | > 32 | (0.5) | - |
| UK | 2 | > 16 | > 16 | > 32 | > 32 | > 16 | 8 | 8 | (0.5) | VIM-4 |
| UK | 0.06 | 16 | 4 | 1 | 16 | 4 | 32 | 4 | (1) | - |
| UK | 0.5 | 16 | 4 | 2 | 16 | 1 | 32 | 4 | (1) | - |
| UK | 0.5 | 16 | 8 | 1 | 16 | 4 | 32 | 8 | (0.5) | - |
| UK | 0.5 | 16 | 8 | 2 | 16 | 4 | 32 | 8 | (0.5) | - |
| UK | 0.06 | > 16 | 16 | 8 | 16 | 4 | 32 | 4 | (1) | - |
| UK | < 0.03 | 16 | 4 | 16 | 8 | 2 | 16 | 16 | (0.5) | PDC‑Type |
| UK | < 0.03 | 16 | 8 | 4 | 16 | 16 | 16 | 32 | (0.5) | - |
| UK | 0.12 | > 16 | 8 | 2 | 32 | > 16 | 32 | 8 | (1) | - |
| UK | 0.06 | > 16 | 8 | 2 | 16 | 8 | 32 | 8 | (0.5) | - |
| UK | 1 | > 16 | 8 | 8 | 16 | 4 | 16 | 8 | (0.5) | PDC‑Type |
| UK | 0.12 | > 16 | 4 | 2 | 8 | 8 | 32 | 8 | (1) | PDC‑19A |
| UK | 0.5 | > 16 | > 16 | 32 | > 32 | 16 | > 32 | > 32 | (1) | - |
| UK | 0.12 | 16 | 4 | 1 | 16 | 4 | 16 | 4 | (0.5) | PDC-Type |
| UK | 2 | > 16 | > 16 | > 32 | > 32 | > 16 | 32 | > 32 | (1) | NDM-1; VEB-9 |
| UK | 2 | 16 | 8 | 8 | 32 | 8 | 32 | 8 | (1) | PDC-Type |
| UK | 0.5 | 16 | 4 | 1 | 16 | 2 | 16 | 8 | (0.5) | PDC-Type |

**B.** Meropenem-resistant *Acinetobacter* spp. isolates (n = 227)

| **Country** | **Organism** | **MIC (mg/L)** | | | | **β-Lactamase genes identified** |
| --- | --- | --- | --- | --- | --- | --- |
|  |  | **FDC** | **MEM** | **SUL-DUR** | **(CST)** |  |
| Austria | *A. baumannii* | 0.25 | > 16 | 2 | (< 0.25) | OXA-23GR |
| Austria | *A. baumannii* | 0.25 | > 16 | 2 | (< 0.25) | OXA-24GR; TEM-1 |
| Austria | *A. baumannii* | 0.25 | > 16 | 1 | (< 0.25) | OXA-23GR |
| Austria | *A. baumannii* | 0.25 | > 16 | 2 | (< 0.25) | OXA-24GR; TEM-1 |
| Austria^a^ | *A. baumannii* | 8 | > 16 | > 16 | (< 0.25) | ADC-74; NDM-1; OXA-66; OXA-72 |
| Austria | *A. baumannii* | 2 | > 16 | 4 | (0.5) | OXA-23GR |
| Austria^b^ | *A. baumannii* | 4 | > 16 | 2 | (< 0.25) | ADC-73; OXA-23-24GR; OXA-66 |
| France | *A. baumannii* | 0.5 | > 16 | 2 | (0.5) | OXA-23GR; TEM-1 |
| France | *A. baumannii* | 0.5 | > 16 | 2 | (< 0.25) | OXA-23-24GR ; TEM-1 |
| France | *A. baumannii* complex | 0.5 | > 16 | 2 | (< 0.25) | OXA-23-24GR ; TEM-1 |
| France | *A. baumannii* | 1 | > 16 | 2 | (< 0.25) | OXA-23-24GR; TEM-1 |
| France | *A. baumannii* | 0.25 | > 16 | 1 | (< 0.25) | OXA-23-24GR; TEM-1 |
| France | *A. baumannii* | 0.5 | > 16 | 2 | (< 0.25) | OXA-23-24GR; TEM-1 |
| France^b^ | *A. baumannii* | 8 | > 16 | 1 | (0.5) | ADC-85; OXA-23GR; OXA-66 |
| France | *A. baumannii* | 1 | 16 | 2 | (< 0.25) | OXA-23GR |
| France | *A. baumannii* | 1 | 16 | 4 | (< 0.25) | OXA-23GR |
| France^b^ | *A. baumannii* | 8 | > 16 | 2 | (< 0.25) | ADC-73; OXA-23GR; OXA-66 |
| France^b^ | *A. baumannii* | 4 | > 16 | 1 | (0.5) | ADC-73; OXA-23GR; OXA-66 |
| France | *A. baumannii* | 0.25 | > 16 | 2 | (< 0.25) | OXA-23GR |
| France | *A. baumannii* complex | 0.25 | > 16 | 1 | (< 0.25) | OXA-23GR |
| France^b^ | *A. baumannii* | 8 | > 16 | 0.5 | (< 0.25) | ADC-204; OXA-23GR; OXA-69; TEM-Trunc |
| France | *A. baumannii* | 32 | > 16 | > 16 | (< 0.25) | ADC-80; NDM-1; OXA-94 |
| France | *A. baumannii* | 0.12 | > 16 | 1 | (0.5) | OXA-23GR |
| France | *A. baumannii* | 2 | > 16 | 0.5 | (< 0.25) | CTX-M-1GR; KPC-3 |
| France^a^ | *A. baumannii* | >32 | > 16 | > 16 | (< 0.25) | ADC-191; NDM-1; OXA-23; OXA-69 |
| France | *A. baumannii* complex | 0.5 | > 16 | 1 | (< 0.25) | OXA-23GR |
| France^a^ | *A. baumannii* | 32 | > 16 | > 16 | (< 0.25) | ADC-191; NDM-1; OXA-23; OXA-69 |
| France | *A. baumannii* | 0.5 | > 16 | 2 | (< 0.25) | OXA-23GR |
| France | *A. baumannii* | 1 | > 16 | 4 | (< 0.25) | OXA-24GR |
| France^a^ | *A. baumannii* | 16 | > 16 | > 16 | (< 0.25) | ADC-165; NDM-1; OXA-94 |
| Germany^a^ | *A. baumannii* | > 32 | > 16 | 8 | (< 0.25) | ADC-73; OXA-23; OXA-66; TEM-1D |
| Germany^a^ | *A. baumannii* | > 32 | > 16 | 1 | (< 0.25) | ADC-30; OXA-23; OXA-66; PER-7 |
| Germany^a^ | *A. baumannii* | 16 | > 16 | > 16 | (< 0.25) | ADC-176; NDM-1; OXA-94 |
| Germany^a^ | *Acinetobacter* spp. | 4 | > 16 | 1 | (< 0.25) | ADC-74; OXA-66; OXA-72 |
| Germany | *A. baumannii* | 0.5 | 16 | 1 | (0.5) | CTX-M-2GR; OXA-24GR |
| Germany | *A. baumannii* | 1 | 16 | 0.5 | (< 0.25) | OXA-23GR |
| Germany^a^ | *A. baumannii* | > 32 | > 16 | 1 | (< 0.25) | ADC-11; OXA-66; OXA-72; PER-1 |
| Italy | *A. baumannii* | 0.5 | > 16 | 4 | (< 0.25) | OXA-23GR; TEM-1 |
| Italy | *A. baumannii* | 0.12 | > 16 | 2 | (< 0.25) | OXA-23GR |
| Italy^a^ | *A. baumannii* | 16 | > 16 | 4 | (< 0.25) | ADC-33; OXA-23; OXA-82 |
| Italy^b^ | *A. baumannii* | 4 | > 16 | 4 | (< 0.25) | ADC-73; OXA-23GR; OXA-66 |
| Italy | *A. baumannii* | 0.25 | > 16 | 2 | (< 0.25) | OXA-23GR |
| Italy | *A. baumannii* | 2 | > 16 | 2 | (< 0.25) | OXA-23GR |
| Italy | *A. baumannii* | 0.25 | > 16 | 1 | (< 0.25) | OXA-23-24GR |
| Italy | *A. baumannii* | 1 | > 16 | 2 | (< 0.25) | OXA-23-24GR; TEM-1 |
| Italy | *A. baumannii* | 1 | > 16 | 1 | (0.5) | OXA-23-24GR |
| Italy | *A. baumannii* | 0.5 | > 16 | 1 | (< 0.25) | OXA-23-24GR |
| Italy | *A. baumannii* | 0.5 | > 16 | 4 | (0.5) | OXA-23GR; TEM-1 |
| Italy | *A. baumannii* | 0.5 | > 16 | 2 | (1) | OXA-23GR; TEM-1 |
| Italy | *A. baumannii* | 0.5 | > 16 | 4 | (< 0.25) | OXA-23GR; TEM-1 |
| Italy | *A. baumannii* | 1 | > 16 | 4 | (< 0.25) | OXA-23GR; TEM-1 |
| Italy | *A. baumannii* | 1 | > 16 | 1 | ( 0.5) | OXA-23GR; TEM-1 |
| Italy | *A. baumannii* | 1 | > 16 | 4 | (< 0.25) | OXA-23GR; TEM-1 |
| Italy | *A. baumannii* | 1 | > 16 | 4 | (< 0.25) | OXA-23GR; TEM-1 |
| Italy | *A. baumannii* | 0.5 | > 16 | 4 | (< 0.25) | OXA-23GR; TEM-1 |
| Italy | *A. baumannii* | 2 | > 16 | 4 | (< 0.25) | OXA-23GR; TEM-1 |
| Italy | *A. baumannii* | 0.5 | 16 | 2 | (< 0.25) | OXA-23GR; TEM-1 |
| Italy | *A. baumannii* | 0.25 | > 16 | 2 | (< 0.25) | OXA-23GR |
| Italy | *A. baumannii* | 2 | > 16 | 2 | (< 0.25) | OXA-23GR; TEM-1 |
| Italy | *A. baumannii* | 2 | > 16 | 4 | (< 0.25) | OXA-23GR; TEM-1 |
| Italy | *A. baumannii* | 2 | > 16 | 4 | (< 0.25) | OXA-23GR; TEM-1 |
| Italy^a^ | *A. baumannii* | 2 | > 16 | 2 | (< 0.25) | ADC-33; OXA-23; OXA-82 |
| Italy | *A. baumannii* | 1 | > 16 | 2 | (0.5) | OXA-23GR; TEM-1 |
| Italy^a^ | *A. baumannii* | 4 | > 16 | 2 | (< 0.25) | ADC-33; OXA-23; OXA-82 |
| Italy^a^ | *A. baumannii* | 2 | > 16 | 4 | (< 0.25) | ADC-33; OXA-23; OXA-82 |
| Italy | *A. baumannii* | 0.5 | > 16 | 4 | (< 0.25) | OXA-23GR; TEM-1 |
| Italy^a^ | *A. baumannii* | 2 | > 16 | 2 | (< 0.25) | ADC-33; OXA-23; OXA-82 |
| Italy^b^ | *A. baumannii* | 16 | > 16 | 1 | (< 0.25) | ADC-33; OXA-23GR; OXA-82 |
| Italy^a^ | *A. baumannii* | 2 | > 16 | 2 | (< 0.25) | ADC-33; OXA-23; OXA-82 |
| Italy | *A. baumannii* | 2 | > 16 | 2 | (< 0.25) | OXA-23GR |
| Italy^a^ | *A. baumannii* | 4 | > 16 | 4 | (< 0.25) | ADC-33; OXA-23; OXA-82 |
| Italy | *A. baumannii* | 0.5 | > 16 | 8 | (< 0.25) | OXA-23GR; TEM-1 |
| Italy^a^ | *A. baumannii* | 2 | > 16 | 2 | (< 0.25) | ADC-33; OXA-23; OXA-82 |
| Italy^a^ | *A. baumannii* | 2 | > 16 | 2 | (< 0.25) | ADC-33; OXA-23; OXA-82 |
| Italy^a^ | *A. baumannii* | 0.5 | > 16 | 4 | (< 0.25) | ADC-73; OXA-23; OXA-66; TEM-1D |
| Italy | *A. baumannii* | 0.5 | > 16 | 4 | (< 0.25) | OXA-23GR; TEM-1 |
| Italy^a^ | *A. baumannii* | > 32 | > 16 | 2 | (8) | ADC-30; OXA-66; OXA-72 |
| Italy^b^ | *A. baumannii* | 16 | > 16 | 2 | (4) | ADC-30; OXA-24GR; OXA-66; OXA-72 |
| Italy^a^ | *A. baumannii* | > 32 | > 16 | 4 | (4) | ADC-30; OXA-66; OXA-72 |
| Italy^b^ | *A. baumannii* | > 32 | > 16 | 2 | (2) | ADC-30; OXA-24GR; OXA-66; OXA-72 |
| Italy | *Acinetobacter* spp. | 0.25 | > 16 | 2 | (< 0.25) | OXA-23GR |
| Italy | *Acinetobacter* spp. | 0.5 | > 16 | 2 | (< 0.25) | CTX-M-2GR; OXA-23-24GR |
| Italy^a^ | *A. baumannii* | 2 | > 16 | 1 | (< 0.25) | ADC-73; OXA-23; OXA-66 |
| Italy^a^ | *A. baumannii* | 2 | > 16 | 2 | (< 0.25) | ADC-73; OXA-23; OXA-66 |
| Italy | *Acinetobacter* spp. | 0.25 | > 16 | 4 | (< 0.25) | OXA-23-24GR |
| Italy | *A. baumannii* | 0.25 | > 16 | 4 | (< 0.25) | OXA-23-24GR |
| Italy | *A. baumannii* | 1 | > 16 | 2 | (< 0.25) | OXA-24GR |
| Italy | *Acinetobacter* spp. | 0.25 | > 16 | 4 | (< 0.25) | OXA-23GR |
| Italy | *A. nosocomialis* | 2 | > 16 | 1 | (< 0.25) | OXA-23GR; TEM-1 |
| Italy | *A. baumannii* | 0.5 | > 16 | 4 | (< 0.25) | OXA-23GR |
| Italy | *Acinetobacter* spp. | 0.25 | > 16 | 4 | (< 0.25) | OXA-23GR |
| Italy | *A. baumannii* | 0.5 | > 16 | 1 | (< 0.25) | OXA-23GR; TEM-1 |
| Italy | *A. baumannii* | 0.25 | > 16 | 4 | (< 0.25) | OXA-23GR |
| Italy | *Acinetobacter* spp. | 0.5 | > 16 | 4 | (< 0.25) | OXA-23GR |
| Italy | *A. baumannii* | 0.5 | > 16 | 2 | (< 0.25) | OXA-23GR |
| Italy | *A. baumannii* complex | 0.25 | > 16 | 2 | (0.5) | OXA-23GR |
| Italy | *A. baumannii* complex | 0.25 | > 16 | 4 | (< 0.25) | OXA-23GR |
| Italy | *A. baumannii* complex | 0.25 | > 16 | 2 | (0.5) | OXA-23GR |
| Italy | *A. baumannii* | 0.25 | 16 | 2 | (0.5) | OXA-23GR |
| Italy | *A. baumannii* complex | 1 | > 16 | 2 | (< 0.25) | OXA-23GR |
| Italy | *A. baumannii* complex | 0.25 | > 16 | 4 | (< 0.25) | OXA-23GR |
| Italy | *A. baumannii* complex | 0.5 | > 16 | 4 | (< 0.25) | OXA-23GR |
| Italy | *A. baumannii* complex | 0.25 | > 16 | 4 | (< 0.25) | OXA-23-24GR |
| Italy | *A. baumannii* complex | 0.5 | > 16 | 4 | (< 0.25) | OXA-23GR |
| Italy | *A. baumannii* complex | 0.25 | > 16 | 4 | (0.5) | OXA-23GR |
| Italy | *A. baumannii* complex | 0.25 | > 16 | 4 | (< 0.25) | OXA-23GR |
| Italy | *A. baumannii* complex | 1 | > 16 | 4 | (< 0.25) | OXA-23GR |
| Italy | *A. baumannii* complex | 0.5 | > 16 | 4 | (0.5) | OXA-23GR |
| Italy | *A. baumannii* | 0.25 | 16 | 2 | (< 0.25) | OXA-23GR |
| Italy^a^ | *A. baumannii* | 16 | > 16 | > 16 | (< 0.25) | ADC-73; NDM-1; OXA-23; OXA-66; TEM-1D |
| Italy^a^ | *A. baumannii* | 16 | > 16 | > 16 | (< 0.25) | ADC-73; NDM-1; OXA-23; OXA-66; TEM-1D |
| Italy^a^ | *Acinetobacter* spp. | 2 | > 16 | 1 | (0.5) | ADC-33; OXA-23; OXA-82 |
| Italy | *Acinetobacter* spp. | 0.25 | > 16 | 2 | (< 0.25) | OXA-23GR |
| Italy | *A. baumannii* | 0.5 | > 16 | 4 | (< 0.25) | OXA-23GR |
| Italy | *Acinetobacter* spp. | 0.5 | > 16 | 1 | (< 0.25) | OXA-23GR |
| Italy^a^ | *A. baumannii* | 16 | > 16 | > 16 | (< 0.25) | ADC-73; NDM-1; OXA-23; OXA-66; TEM-1D |
| Italy | *A. baumannii* | 0.25 | > 16 | 2 | (0.5) | OXA-23GR |
| Italy^a^ | *A. baumannii* | 8 | > 16 | > 16 | (< 0.25) | ADC-73; NDM-1; OXA-23; OXA-66; TEM-1D |
| Italy^a^ | *A. baumannii* | 8 | > 16 | > 16 | (< 0.25) | ADC-Trunc; NDM-1; OXA-23; OXA‑66; TEM-1D |
| Italy | *Acinetobacter* spp. | 0.5 | > 16 | 4 | (< 0.25) | OXA-23GR; TEM-1 |
| Italy | *A. baumannii* | 1 | > 16 | 2 | (< 0.25) | OXA-23GR |
| Italy^a^ | *Acinetobacter* spp. | 16 | > 16 | > 16 | (< 0.25) | ADC-73; NDM-1; OXA-23; OXA-66; TEM-1D |
| Italy | *A. baumannii* | 0.5 | > 16 | 2 | (< 0.25) | OXA-23GR; TEM-1 |
| Italy | *A. baumannii* complex | 0.5 | > 16 | 1 | (< 0.25) | OXA-23GR |
| Italy | *A. baumannii* | 0.5 | > 16 | 1 | (0.5) | OXA-23GR |
| Italy | *A. baumannii* | 1 | > 16 | 1 | (< 0.25) | OXA-23GR |
| Italy | *A. baumannii* complex | 0.5 | > 16 | 1 | (0.5) | OXA-23GR |
| Italy | *A. baumannii* | 1 | > 16 | 1 | (< 0.25) | OXA-23GR |
| Italy | *A. baumannii* | 0.25 | > 16 | 4 | (< 0.25) | OXA-23GR; TEM-1 |
| Italy | *A. baumannii* | 1 | > 16 | 4 | (< 0.25) | OXA-23GR; TEM-1 |
| Italy | *A. baumannii* | 1 | > 16 | 1 | (< 0.25) | OXA-23GR |
| Italy | *A. baumannii* | 2 | 16 | 1 | (< 0.25) | OXA-23GR |
| Italy | *A. baumannii* | 2 | > 16 | 1 | (< 0.25) | OXA-23GR |
| Italy | *A. baumannii* complex | 0.5 | > 16 | 4 | (< 0.25) | OXA-23GR; TEM-1 |
| Italy | *A. baumannii* | 0.5 | > 16 | 2 | (< 0.25) | OXA-23GR |
| Italy | *A. baumannii* | 0.5 | > 16 | 1 | (< 0.25) | OXA-23GR |
| Italy | *A. baumannii* | 0.5 | > 16 | 1 | (0.5) | OXA-23GR |
| Italy | *A. baumannii* | 2 | > 16 | 2 | (0.5) | OXA-23GR |
| Italy^a^ | *A. baumannii* | 2 | 16 | 1 | (2) | ADC-33; OXA-23; OXA-82 |
| Italy^a^ | *A. baumannii* | 2 | 16 | 0.5 | (0.5) | ADC-33; OXA-23; OXA-82 |
| Italy^a^ | *A. baumannii* | 4 | 16 | 0.5 | (2) | ADC-33; OXA-23; OXA-82 |
| Italy^a^ | *A. baumannii* | 4 | 16 | 1 | (0.5) | ADC-33; OXA-23; OXA-82 |
| Italy | *A. baumannii* complex | 2 | >16 | 1 | (< 0.25) | OXA-23GR |
| Italy | *A. baumannii* complex | 0.5 | > 16 | 2 | (< 0.25) | OXA-23GR; TEM-1 |
| Italy | *A. baumannii* complex | < 0.03 | > 16 | 1 | (0.5) | OXA-24GR |
| Italy^a^ | *A. baumannii* complex | 1 | 16 | 1 | (0.5) | ADC-33; OXA-82 |
| Italy | *A. baumannii* complex | 0.5 | > 16 | 2 | (< 0.25) | OXA-23GR |
| Italy | *A. baumannii* complex | 0.5 | >16 | 2 | (< 0.25) | OXA-23GR |
| Italy | *A. baumannii* complex | < 0.03 | 16 | 1 | (0.5) | - |
| Italy | *A. baumannii* complex | 0.25 | > 16 | 2 | (0.5) | OXA-23GR; TEM-1 |
| Italy | *A. baumannii* complex | 2 | > 16 | 1 | (0.5) | OXA-23GR |
| Italy | *A. baumannii* | 0.25 | 16 | 1 | (< 0.25) | - |
| Italy | *A. baumannii* | 0.5 | > 16 | 1 | (< 0.25) | OXA-23GR; TEM-1 |
| Italy | *A. baumannii* | 0.5 | > 16 | 1 | (0.5) | OXA-23GR |
| Italy | *A. baumannii* | 0.5 | > 16 | 1 | (0.5) | OXA-23GR; TEM-1 |
| Italy | *A. baumannii* | 0.5 | > 16 | 1 | (0.5) | OXA-23GR; TEM-1 |
| Italy | *A. baumannii* | 0.5 | > 16 | 1 | (< 0.25) | OXA-23GR; TEM-1 |
| Italy | *A. baumannii* | 0.5 | > 16 | 0.5 | (< 0.25) | OXA-23GR; TEM-1 |
| Italy | *A. baumannii* | 2 | > 16 | 1 | (>8) | OXA-23GR |
| Italy | *A. baumannii* | 0.5 | > 16 | 1 | (0.5) | OXA-23-24GR; TEM-1 |
| Italy | *A. baumannii* | 0.5 | > 16 | 2 | (0.5) | OXA-23GR; TEM-1 |
| Italy | *A. baumannii* | 0.5 | >16 | 1 | (0.5) | OXA-23GR; TEM-1 |
| Italy | *A. baumannii* complex | 0.5 | >16 | 1 | (< 0.25) | OXA-23GR; TEM-1 |
| Italy | *A. baumannii* | 0.5 | >16 | 1 | (1) | OXA-23-58GR; TEM-1 |
| Italy | *A. baumannii* | 0.5 | >16 | 1 | (0.5) | OXA-23GR; TEM-1 |
| Italy | *A. baumannii* complex | 0.25 | >16 | 0.5 | (0.5) | OXA-23GR; TEM-1 |
| Italy | *A. baumannii* | 1 | >16 | 4 | (< 0.25) | OXA-23GR |
| Italy | *A. baumannii* complex | 0.25 | >16 | 4 | (< 0.25) | OXA-23GR |
| Italy | *A. baumannii* complex | 0.5 | >16 | 4 | (< 0.25) | OXA-24GR; TEM-1 |
| Italy | *A. baumannii* | 0.5 | >16 | 4 | (0.5) | OXA-23GR; TEM-1 |
| Italy | *A. baumannii* complex | 0.5 | >16 | 4 | (< 0.25) | OXA-23GR; TEM-1 |
| Italy | *A. baumannii* complex | 0.5 | >16 | 4 | (< 0.25) | OXA-23GR |
| Spain | *A. baumannii* | 0.06 | >16 | 1 | (< 0.25) | OXA-23GR |
| Spain | *A. baumannii* | 0.25 | >16 | 1 | (< 0.25) | OXA-23GR |
| Spain | *A. baumannii* | 0.5 | >16 | 2 | (< 0.25) | OXA-23GR |
| Spain | *A. baumannii* | 0.12 | >16 | 1 | (0.5) | OXA-23GR |
| Spain | *A. baumannii* | 0.25 | >16 | 1 | (< 0.25) | OXA-23GR |
| Spain^a^ | *A. baumannii* | > 32 | >16 | 1 | (0.5) | ADC-30; OXA-23; OXA-66 |
| Spain | *A. baumannii* | 0.25 | >16 | 1 | (0.5) | OXA-23GR |
| Spain | *A. baumannii* | 0.12 | >16 | 1 | (< 0.25) | OXA-23GR |
| Spain^a^ | *A. baumannii* | 0.5 | >16 | 1 | (0.5) | ADC-30; OXA-23; OXA-66 |
| Spain^a^ | *A. baumannii* | 0.5 | >16 | 1 | (0.5) | ADC-30; OXA-23; OXA-66 |
| Spain | *A. baumannii* | 0.25 | >16 | 2 | (< 0.25) | OXA-24GR |
| Spain | *A. baumannii* | 0.12 | >16 | 1 | (0.5) | OXA-23GR |
| Spain | *A. baumannii* | 0.12 | >16 | 1 | (0.5) | OXA-23GR |
| Spain | *A. baumannii* | 0.12 | >16 | 1 | (0.5) | OXA-23GR |
| Spain | *A. baumannii* | 0.25 | 16 | 2 | (< 0.25) | - |
| Spain | *A. baumannii* | 0.12 | >16 | 2 | (< 0.25) | - |
| Spain | *A. baumannii* | 0.5 | >16 | 0.5 | (< 0.25) | - |
| Spain | *A. baumannii* | 0.5 | >16 | 2 | (< 0.25) | - |
| Spain | *A. baumannii* | 0.25 | 16 | 2 | (0.5) | - |
| Spain | *A. baumannii* | 0.5 | >16 | 0.5 | (0.5) | OXA-23GR |
| Spain | *A. baumannii* | 0.25 | >16 | 1 | (< 0.25) | OXA-23GR |
| Spain | *A. baumannii* | 0.12 | > 16 | 1 | (< 0.25) | OXA-23GR |
| Spain | *A. baumannii* | 0.25 | > 16 | 1 | (< 0.25) | OXA-23GR |
| Spain | *A. baumannii* | 0.25 | > 16 | 1 | (< 0.25) | OXA-23GR |
| Spain | *A. baumannii* | 2 | > 16 | 1 | (> 8) | OXA-24GR |
| Spain | *A. baumannii* | 0.5 | > 16 | 2 | (< 0.25) | OXA-23GR |
| Spain | *A. baumannii* | 0.5 | > 16 | 2 | (< 0.25) | OXA-23GR |
| Spain | *A. baumannii* | 0.25 | > 16 | 2 | (< 0.25) | OXA-23GR |
| Spain | *A. baumannii* | 1 | > 16 | 4 | (< 0.25) | OXA-23GR |
| Spain | *A. baumannii* | 0.5 | > 16 | 2 | (< 0.25) | OXA-23GR |
| Spain | *A. bereziniae* | 0.12 | > 16 | < 0.25 | (0.5) | OXA-24GR |
| Spain | *A. baumannii* | 0.25 | > 16 | 2 | (< 0.25) | OXA-23GR |
| Spain | *A. baumannii* | 1 | > 16 | 2 | (< 0.25) | OXA-23GR |
| Spain | *A. baumannii* | 0.5 | > 16 | 1 | (< 0.25) | OXA-23GR |
| Spain^a^ | *A. baumannii* | 0.25 | > 16 | 1 | (< 0.25) | ADC-30; OXA-23; OXA-66 |
| Spain | *A. baumannii* | 1 | > 16 | 1 | (< 0.25) | OXA-23GR |
| Spain | *A. baumannii* | 0.5 | > 16 | 1 | (< 0.25) | OXA-23GR |
| Spain | *A. baumannii* | 0.5 | > 16 | 2 | (0.5) | OXA-23GR |
| Spain^a^ | *A. baumannii* | 2 | > 16 | 1 | (< 0.25) | ADC-30; OXA-23; OXA-66 |
| Spain | *A. baumannii* | 0.5 | > 16 | 1 | (< 0.25) | OXA-23GR |
| Spain | *A. baumannii* | 0.25 | > 16 | 1 | (< 0.25) | OXA-23GR |
| Spain | *A. baumannii* | 0.25 | > 16 | 1 | (< 0.25) | OXA-23GR |
| Spain | *A. baumannii* | 0.5 | > 16 | 1 | (< 0.25) | OXA-23GR |
| Spain | *A. baumannii* | 0.25 | > 16 | 1 | (< 0.25) | OXA-23GR |
| Spain | *A. baumannii* | 0.5 | > 16 | 1 | (0.5) | OXA-23GR |
| Spain | *A. baumannii* | 0.25 | > 16 | 1 | (< 0.25) | OXA-23GR |
| Spain | *A. baumannii* | 0.5 | > 16 | 1 | (0.5) | OXA-24GR |
| Spain | *A. baumannii* | 0.25 | > 16 | 2 | (< 0.25) | OXA-24GR |
| Spain | *A. baumannii* | 1 | > 16 | 2 | (< 0.25) | OXA-24GR |
| Spain | *A. baumannii* | 0.25 | > 16 | 2 | (< 0.25) | OXA-24GR |
| Spain | *A. baumannii* complex | 0.25 | > 16 | 2 | (< 0.25) | OXA-24GR |
| UK^a^ | *A. baumannii* | 2 | > 16 | 2 | (> 8) | ADC-294; OXA-23; OXA-66 |
| UK | *Acinetobacter* spp. | 0.25 | > 16 | 2 | (< 0.25) | OXA-23GR; TEM-1 |
| UK^a^ | *Acinetobacter* spp. | > 32 | > 16 | 2 | (0.5) | ADC-73; OXA-23; OXA-66; TEM-1D |
| UK^a^ | *A. baumannii* | 0.5 | > 16 | 1 | (1) | ADC-73; OXA-23; OXA-66 |

^a^ Cefiderocol-resistant isolates with β-lactamase gene data from WGS.

^b^ Isolates with β-lactamase gene data collated from PCR and WGS.

ADC, *Acinetobacter*-derived cephalosporinase; AmpC, ampicillinase C; ATM-AVI, aztreonam-avibactam; CST, colistin; C/T, ceftolozane/tazobactam; CZA, ceftazidime-avibactam; ESBL, extended-spectrum β-lactamase; FDC, cefiderocol; FEP-TAN, cefepime-taniborbactam; GES, Guiana extended‑spectrum β‑lactamase; GR, group; KPC, *Klebsiella pneumoniae* carbapenemase; I-R, imipenem-relebactam; MBL, metallo-β-lactamase; MEM, meropenem; MVB, meropenem-vaborbactam; NDM, New Delhi MBL; OXA, oxacillinase; PCR, polymerase chain reaction; PDC, *Pseudomonas*-derived cephalosporinase; UK, United Kingdom; VIM, Verona integron-borne metallo-β-lactamase; WGS, whole genome sequencing, Y, yes.

β-Lactamase data were generated from PCR unless otherwise indicated. Colistin data are shown in parentheses as colistin is not recommended for monotherapy and is not associated with a clinical monotherapy breakpoint (as per EUCAST v.14.0 guidance).

**TABLE S7** Participating sites

| **Country** | **Centre** | **Lead investigator** |
| --- | --- | --- |
| Austria | AKH Wien | Birgit Willinger |
| France | CH de la Côte Basque | David Leyssene |
| France | CH de Valenciennes | Christian Cattoen |
| France | CHRU de Nancy - Hôpital Brabois Adultes | Corentine Alauzet |
| France | CHRU de Strasbourg | Pierre Boyer |
| France | CHU de Bordeaux | Véronique Dubois |
| France | CHU de Besançon - Hôpital Jean-Minjoz | Katy Jeannot |
| France | CHU de Nantes | Stephane Corvec |
| France | CHU de Nîmes | Jean-Philippe Lavigne |
| France | CHU de Reims | Thomas Guillard |
| France | HIA Bégin | Audrey Merens Gontier |
| France | Hôpital Bicêtre | Thierry Naas |
| Germany | Universitätsklinikum Hamburg-Eppendorf | Holger Rohde |
| Germany | Medizinische Hochschule Hannover | Stefan Ziesing |
| Germany | Justus-Liebig-Universität Giessen | Can Imirzalioglu |
| Germany | Krankenhaus Nordwest Zentralinstitut für Laboratoriumsmedizin | Klaus-Peter Hunfeld |
| Germany | LMU München | Jette Jung |
| Germany | Ruhr-Universität Bochum | Sören Gatermann |
| Germany | Universitatsklinikum Jena | Mathias Pletz |
| Italy | AOU Città della Salute e della Scienza di Torino | Gabriele Bianco |
| Italy | AOU Policlinico Paolo Giaccone | Anna Giammanco |
| Italy | ASST Monza - Ospedale di Desio | Davide Carcione |
| Italy | Azienda Policlinico Umberto I di Roma | Giammarco Raponi |
| Italy | Fondazione IRCCS Ca’ Granda Ospedale Maggiore Policlinico | Caterina Matinato |
| Italy | Istituti Fisioterapici Ospitalieri | Enea Gino Di Domenico |
| Italy | IRCCS Policlinico di Sant’Orsola-Malpighi | Paolo Gaibani |
| Italy | IRCCS Ospedale Policlinico San Martino | Anna Marchese |
| Italy | Università degli studi di Foggia | Fabio Arena |
| Italy | Università degli Studi di Firenze | Claudia Niccolai |
| Italy | Università degli Studi di Catania | Stefania Stefani |
| Spain | Hospital Clínic de Barcelona | Cristina Pitart |
| Spain | Hospital Universitario Cruces | Jose Luis Barrios |
| Spain | Hospital General Universitario Gregorio Marañón | Emilia Cercenado |
| Spain | Hospital Universitario de A Coruña | German Bou |
| Spain | Hospital Nuestra Señora del Prado | Alicia Beteta Lopez |
| Spain | Hospital Universitario Ramón y Cajal | Rafael Canton |
| Spain | Hospital Universitario y Politécnico La Fe | Jose Lopez Hontangas |
| Spain | Hospital Universitario Reina Sofía | Irene Gracia-Ahufinger |
| Spain | Hospital Universitario Son Espases | Antonio Oliver |
| Spain | Hospital Universitario Virgen Macarena | Lorena Lopez-Cerero |
| Spain | Hospital Vall d’Hebron | Nieves Larrosa |
| UK | Queen Mary University of London | David Wareham |
| UK | Freeman Hospital, Newcastle Hospitals NHS Foundation Trust | John Perry |
| UK | Queen Elizabeth Hospital Birmingham, University Hospitals Birmingham NHS Foundation Trust | Anna Casey |
| UK | Leeds Teaching Hospitals NHS Trust | Jasvir Nahl |
| UK | Manchester University Hospital, NHS Foundation Trust | Daniel Hughes |
| UK | Glasgow Royal Infirmary, NHS Greater Glasgow and Clyde | Michael Coyne |
| UK | Queen’s Medical Centre, Nottingham University Hospitals NHS Trust | Michelle Lister |
| UK | Southmead Hospital, North Bristol NHS Trust | Marie Attwood |

AKH Wein, Allgemeines Krankenhaus der Stadt Wien; AOU, Azienda Ospedaliera Universitaria; CH, Centre Hospitalier; CHU, CH Universitaire; CHRU, CH Régional Universitaire; HIA, L'hôpital d'instruction des armées; IRCCS, Istituto di Ricovero e Cura a Carattere Scientifico; LMU, Ludwig-Maximilians-Universität; NHS, National Health Service.

**TABLE S8** Suppliers of antimicrobial agents

| **Agent** | **Supplier** | **Product code** |
| --- | --- | --- |
| Aztreonam | USP (North Bethesda, MA, USA) | 1046205 |
| Cefepime hydrochloride | Sigma-Aldrich (St. Louis, MO, USA) | PHR1763 |
| Cefiderocol | AVEP (IHMA 3825) | N/A |
| Ceftolozane sulfate | TOKU-E (Bellingham, WA, USA) | C245 |
| Colistin sulfate | USP | 1148001 |
| Durlobactam sodium | AdooQ Bioscience (Irvine, CA, USA) | A13801 |
| Imipenem monohydrate | USP | 1337809 |
| Meropenem | USP | 1392454 |
| Relebactam | MedChemExpress (Monmouth Junction, NJ, USA) | HY-16752 |
| Sulbactam | USP | 1623670 |
| Taniborbactam | MedChemExpress | 2244235-49-0 |
| Tazobactam | Selleckchem (Houston, TX, USA) | S3077 |
| Vaborbactam | MedChemExpress | HY-19930 |

AVEP, Antimicrobial Voluntary Evaluation Program; IHMA, International Health Management Associates; N/A, not applicable; USA, United States of America; USP, United States pharmacopeia.

Excludes avibactam and ceftazidime, as Sensititre™ freeze dried panels (Thermo Fisher Scientific Inc., Waltham, MA, USA) were used in the preparation of ceftazidime‑avibactam for testing.

**TABLE S9** Antimicrobial susceptibility testing MIC breakpoints for *P. aeruginosa* and *Acinetobacter* spp.

1. EUCAST breakpoints (v.14.0, 2024)^1^

| **Antimicrobial** | **Organism** | **MIC breakpoint (mg/L)** | |
| --- | --- | --- | --- |
|  |  | **S or I (≤)** | **R (>)** |
| Cefiderocol | *P. aeruginosa* | 2 | 2 |
|  | *Acinetobacter* spp.^a^ | (2) | (2) |
| Meropenem^b^ | *P. aeruginosa* | 8 | 8 |
|  | *Acinetobacter* spp. | 8 | 8 |
| Ceftazidime-avibactam | *P. aeruginosa*^c^ | 8 | 8 |
|  | *Acinetobacter* spp. | - | - |
| Ceftolozane/tazobactam | *P. aeruginosa*^c^ | 4 | 4 |
|  | *Acinetobacter* spp. | - | - |
| Meropenem-vaborbactam^d^ | *P. aeruginosa*^c^ | 8 | 8 |
|  | *Acinetobacter* spp. | - | - |
| Imipenem-relebactam | *P. aeruginosa*^c^ | 2 | 2 |
|  | *Acinetobacter* spp.^c^ | 2 | 2 |
| Aztreonam-avibactam^b^ | *P. aeruginosa*^d^ | 16 | 16 |
|  | *Acinetobacter* spp. | - | - |
| Cefepime-taniborbactam^b^ | *P. aeruginosa*^d^ | 8 | 8 |
|  | *Acinetobacter* spp. | - | - |
| (Colistin)^e^ | *P. aeruginosa* | (4) | (4) |
|  | *Acinetobacter* spp. | (2) | (2) |

1. CLSI or FDA breakpoints (2023)^2^

| **Antimicrobial** | **Organism** | **MIC breakpoint (mg/L)** | | |
| --- | --- | --- | --- | --- |
|  |  | **S (≤)** | **I** | **R (≥)** |
| Cefiderocol | *P. aeruginosa* | 4 | 8 | 16 |
|  | *Acinetobacter* spp. | 4 | 8 | 16 |
| Meropenem | *P. aeruginosa* | 2 | 4 | 8 |
|  | *Acinetobacter* spp. | 2 | 4 | 8 |
| Ceftazidime-avibactam^d^ | *P. aeruginosa* | 8/4 | - | 16/4 |
|  | *Acinetobacter* spp. | 8 | 16 | 32 |
| Ceftolozane/tazobactam | *P. aeruginosa* | 4/4 | 8/4 | 16/4 |
|  | *Acinetobacter* spp. | - | - | - |
| Meropenem-vaborbactam^d^ | *P. aeruginosa* | 2 | 4 | 8 |
|  | *Acinetobacter* spp. | 2 | 4 | 8 |
| Imipenem-relebactam^d^ | *P. aeruginosa* | 2/4 | 4/4 | 8/4 |
|  | *Acinetobacter* spp. | 2 | 4 | 8 |
| Aztreonam-avibactam | *P. aeruginosa* | 8 | 16 | 32 |
|  | *Acinetobacter* spp. | - | - | - |
| Cefepime-taniborbactam^d^ | *P. aeruginosa* | 8 | 16 | 32 |
|  | *Acinetobacter* spp. | 8 | 16 | 32 |
| Sulbactam-durlobactam^f^ | *P. aeruginosa* | - | - | - |
|  | *Acinetobacter* spp. | 4 | - | 16 |
| (Colistin) | *P. aeruginosa* | - | ≤ 2 | 4 |
|  | *Acinetobacter* spp. | - | ≤ 2 | 4 |

^a^ Non-species-specific PK/PD breakpoint shown in parentheses.

^b^ High dosage breakpoints.

^c^ For susceptibility testing purposes, the concentrations of avibactam, tazobactam and relebactam were fixed at 4 mg/L, and the concentration of vaborbactam was fixed at 8 mg/L.

^d^ Breakpoints for the agent without inhibitor.

^e^ Colistin is not recommended for monotherapy and is not associated with a clinical monotherapy breakpoint (as per EUCAST v.14.0 guidance); epidemiological cut-off values were used and are shown in parentheses.^1,3^

^f^ FDA breakpoint was used.^4^

CLSI, Clinical and Laboratory Standards Institute; EUCAST, European Committee on Antimicrobial Susceptibility Testing; FDA, Food and Drug Administration; I, susceptible, increased exposure; PD, pharmacodynamic; PK, pharmacokinetic; R, resistant; S, susceptible.

Breakpoints used to analyze isolates collected in this study were those recommended by EUCAST, with the exception of the FDA MIC breakpoint for sulbactam‑durlobactam.

**TABLE S10** Polymerase chain reaction primer sequences used for detection of β-lactamase genes in *P. aeruginosa* and *Acinetobacter* spp. isolates

| **Target gene** | **Primer name** | **Primer sequence 5'-3'** | **Annealing temperature, °C** |
| --- | --- | --- | --- |
| TEM | TEM-3 | CATTTCCGTGTCGCCCTTATTC | 59 |
|  | TEM-4 | CGTTCATCCATAGTTGCCTGAC |  |
| SHV | SHV-5 | CCTTTAAAGTAGTGCTCTGC | 59 |
|  | SHV-6 | TTCGCTGACCGGCGAGTAGT |  |
| VEB | VEB-F | CATTTCCCGATGCAAAGCGT | 59 |
|  | VEB-R | CGAAGTTTCTTTGGACTCTG |  |
| PER | PERpan-F | TAGGYGTTGCMGTRTGGGG | 59 |
|  | PERpan-R | GGTTTCRACCATCCAYTTCC |  |
| GES | GES-F | AGTCGGCTAGACCGGAAAG | 59 |
|  | GES-R | TTTGTCCGTGCTCAGGAT |  |
| OXA-24 group (only for *P. aeruginosa*) | OXA-24-F | GGTTAGTTGGCCCCCTTAAA | 59 |
|  | OXA-24-R | AGTTGAGCGAAAAGGGGATT |  |
| OXA-23 group (only for *Acinetobacter* spp.) | OXA-23-F | GATCGGATTGGAGAACCAGA | 59 |
|  | OXA-23-R | ATTTCTGACCGCATTTCCAT |  |
| OXA-24 group (only for *Acinetobacter* spp.) | OXA-24-F | GGTTAGTTGGCCCCCTTAAA | 59 |
|  | OXA-24-R | AGTTGAGCGAAAAGGGGATT |  |
| OXA-58 group (only for *Acinetobacter* spp.) | OXA-58-F | AAGTATTGGGGCTTGTGCTG | 59 |
|  | OXA-58-R | CCCCTCTGCGCTCTACATAC |  |
| 16S | 16s-519r | GWATTACCGCGGCKGCTG | 59 |
|  | U341F | CCT ACG GGR SGC AGC AG |  |
| IMP | IMP2-F | GGAATAGAGTGGCTTAAYTCTC | 59 |
|  | IMP2r4 | GGTTTAAYAAARCAACCRCC |  |
| VIM | VIM-F | GATGGTGTTTGGTCGCATA | 59 |
|  | VIM-R3 | CGAATGCGCAGCACCAGGA |  |
| SPM | SPM-F | AAAATCTGGGTACGCAAACG | 59 |
|  | SPM-R | ACATTATCCGCTGGAACAGG |  |
| NDM | NDM-F | CCGTATGAGTGATTGCGGCG | 59 |
|  | NDM-R | GCCCAATATTATGCACCCGG |  |
| KPC | KPCy-F | TGTCACTGTATCGCCGTC | 59 |
|  | KPCy-R | CTCAGTGCTCTACAGAAAACC |  |
| GIM | GIM-F | TCGACACACCTTGGTCTGAA | 59 |
|  | GIM-R | AACTTCCAACTTTGCCATGC |  |
| PDC (only for *P. aeruginosa*) | PDC-123-F | CGTRCAACCGGTSATGAAGGC | 59 |
|  | PDC-273-R | CGGTGAAGGTCTTGCTCAC |  |
| MOX | MOX-MF | GCTGCTCAAGGAGCACAGGAT | 65 |
|  | MOX-MR | CACATTGACATAGGTGTGGTGC |  |
| FOX | FOX-MF | AACATGGGGTATCAGGGAGATG | 65 |
|  | FOX-MR | CAAAGCGCGTAACCGGATTGG |  |
| ACC | ACC-MF | AACAGCCTCAGCAGCCGGTTA | 65 |
|  | ACC-MR | TTCGCCGCAATCATCCCTAGC |  |
| CMY II | CIT-MF | TGGCCAGAACTGACAGGCAAA | 65 |
|  | CIT-MR | TTTCTCCTGAACGTGGCTGGC |  |
| ACT/MIR | EBC-MF | TCGGTAAAGCCGATGTTGCGG | 65 |
|  | EBC-MR | CTTCCACTGCGGCTGCCAGTT |  |
| DHA | DHA-MF | AACTTTCACAGGTGTGCTGGGT | 65 |
|  | DHA-MR | CCGTACGCATACTGGCTTTGC |  |
| CTX-M-1 | CTX-M1f2 | AAAAATCACTGCGCCAGTTC | 58 |
|  | CTX-M1r2 | AGCTTATTCATCGCCACGTT |  |
| CTX-M-2 | CTX-M2f2 | CGACGCTACCCCTGCTATT | 58 |
|  | CTX-M2r2 | CCAGCGTCAGATTTTTCAGG |  |
| CTX-M-9 | CTX-M9f2 | CAAAGAGAGTGCAACGGATG | 58 |
|  | CTX-M9r2 | ATTGGAAAGCGTTCATCACC |  |
| CTX-M-8 | CTX-M-8A | TCGCGTTAAGCGGATGATGC | 58 |
|  | CTX-M-8-25B | AACCCACGATGTGGGTAGC |  |
| CTX-M-25 | CTX-M-25A | GCACGATGACATTCGGG | 58 |
|  | CTX-M-8-25B | AACCCACGATGTGGGTAGC |  |

ACC, Ambler Class C; F, forward primer; GES, Guiana extended-spectrum β-lactamase; GIM, German imipenemase; IMP, imipenemase; KPC, *Klebsiella pneumoniae* carbapenemase; MBL, metallo-β-lactamase; MF, modified F; MR, modified R; NDM, New Delhi MBL; OXA, oxacillinase; PDC, *Pseudomonas* derived cephalosporinase; PER, *Pseudomonas* extended resistant; R, reverse primer; VEB, Vietnamese extended-spectrum β-lactamase; VIM, Verona integron-borne MBL.

**TABLE S11** Species-specific reference genes used for whole genome sequencing of cefiderocol-resistant *P. aeruginosa* and *Acinetobacter* spp. isolates

| **Species** | **Strain** | **Gene name** | **Nucleotide accession** | **Locus tag** | **Amino acid accession** |
| --- | --- | --- | --- | --- | --- |
| *P. aeruginosa* | PAO1 | *ftsI* | NC_002516 | PA4418 | NP_253108.1 |
|  |  | *oprD* |  | PA0958 | NP_249649.1 |
|  |  | *piuA* |  | PA4514 | NP_253204.1 |
|  |  | *piuC* |  | PA4515 | NP_253205.1 |
|  |  | *pirA* |  | PA0931 | NP_249622.1 |
|  |  | *pvdS* |  | PA2426 | NP_251116.1 |
| *A. baumannii* | ATCC 19606 | *ftsI* | NZ_MJHA010000^a^ | BIT33_RS12135 | WP_000227936.1 |
|  |  | *carO* |  | BIT33_RS07010 | WP_000866529.1 |
|  |  | *piuA* |  | BIT33_RS10190 | WP_001984473.1 |
|  |  | *piuC* |  | BIT33_RS10185 | WP_001984475.1 |
|  |  | *pirA* |  | BIT33_RS15810 | WP_000044170.1 |

^a^ Reference sequence is truncated through a nonsense mutation; the full-length protein is considered to be 373 amino acids.

**SUPPLEMENTARY REFERENCES**

1. European Committee on Antimicrobial Susceptibility Testing, EUCAST. Breakpoint tables for interpretation of MICs and zone diameters, Version 14.0. 2024. Available from: https://www.eucast.org/fileadmin/src/media/PDFs/EUCAST_files/Breakpoint_tables/v_14.0_Breakpoint_Tables.pdf. Accessed: February 2024.
2. Clinical and Laboratory Standards Institute, CLSI. M100: Performance Standards for Antimicrobial Susceptibility Testing. 33rd Edition. 2023. Available from: https://clsi.org/standards/products/microbiology/documents/m100/. Accessed: February 2024.
3. European Committee on Antimicrobial Susceptibility Testing, EUCAST. Breakpoint Committee consultation on Colistin. *Colistin Breakpoints - Guidance document 2022.*
4. United States Food and Drug Administration. Sulbactam and Durlobactam Injection. Available at: https://www.fda.gov/drugs/development-resources/sulbactam-and-durlobactam-injection. Accessed: February 2024.
